# Comparing the performance potential of speckle contrast optical spectroscopy and diffuse correlation spectroscopy for cerebral blood flow monitoring using Monte Carlo simulations in realistic head geometries

**DOI:** 10.1117/1.NPh.11.1.015004

**Published:** 2024-01-27

**Authors:** Mitchell B. Robinson, Tom Y. Cheng, Marco Renna, Melissa M. Wu, Byungchan Kim, Xiaojun Cheng, David A. Boas, Maria Angela Franceschini, Stefan A. Carp

**Affiliations:** a Massachusetts General Hospital, Harvard Medical School, Athinoula A. Martinos Center for Biomedical Imaging, Department of Radiology, Boston, Massachusetts, United States; b Boston University, Neurophotonics Center, Department of Biomedical Engineering, Boston, Massachusetts, United States; c Duke University, Department of Biomedical Engineering, Durham, North Carolina, United States

**Keywords:** diffuse correlation spectroscopy, speckle contrast optical spectroscopy, Monte Carlo simulation, cerebral blood flow

## Abstract

**Significance:**

The non-invasive measurement of cerebral blood flow based on diffuse optical techniques has seen increased interest as a research tool for cerebral perfusion monitoring in critical care and functional brain imaging. Diffuse correlation spectroscopy (DCS) and speckle contrast optical spectroscopy (SCOS) are two such techniques that measure complementary aspects of the fluctuating intensity signal, with DCS quantifying the temporal fluctuations of the signal and SCOS quantifying the spatial blurring of a speckle pattern. With the increasing interest in the use of these techniques, a thorough comparison would inform new adopters of the benefits of each technique.

**Aim:**

We systematically evaluate the performance of DCS and SCOS for the measurement of cerebral blood flow.

**Approach:**

Monte Carlo simulations of dynamic light scattering in an MRI-derived head model were performed. For both DCS and SCOS, estimates of sensitivity to cerebral blood flow changes, coefficient of variation of the measured blood flow, and the contrast-to-noise ratio of the measurement to the cerebral perfusion signal were calculated. By varying complementary aspects of data collection between the two methods, we investigated the performance benefits of different measurement strategies, including altering the number of modes per optical detector, the integration time/fitting time of the speckle measurement, and the laser source delivery strategy.

**Results:**

Through comparison across these metrics with simulated detectors having realistic noise properties, we determine several guiding principles for the optimization of these techniques and report the performance comparison between the two over a range of measurement properties and tissue geometries. We find that SCOS outperforms DCS in terms of contrast-to-noise ratio for the cerebral blood flow signal in the ideal case simulated here but note that SCOS requires careful experimental calibrations to ensure accurate measurements of cerebral blood flow.

**Conclusion:**

We provide design principles by which to evaluate the development of DCS and SCOS systems for their use in the measurement of cerebral blood flow.

## 1 Introduction

Diffuse correlation spectroscopy (DCS), an optical technique that relies on sampling the intensity fluctuations of a speckle pattern set-up by diffusely scattered coherent light to quantify deep tissue blood flow, is seeing increased adoption as a non-invasive research tool for cerebral perfusion monitoring in clinical settings including critical care and sleep apnea, and for functional brain imaging.^1^^–^^6^ However, as usually implemented, DCS relies on the use of single or few-mode fibers to sample the speckle pattern in conjunction with photon counting detectors to construct the intensity temporal auto-correlation function, $g_{2}(\tau )$.^7^ This dramatically limits the photon throughput [e.g., in comparison with near-infrared spectroscopy (NIRS) where large area detectors and multi-mode fiber bundles can be used] and poses practical challenges to the use of DCS in translational studies. Consequently, substantial effort in the field is now dedicated to improving the signal-to-noise ratio (SNR) of diffuse optical blood flow measurements.^8^

In this context, a major area of investigation is focused on the use of massively parallel detection. This can be achieved to some extent by employing single photon avalanche photodiode (SPAD) arrays.^9^^–^^11^ However, a more practical approach is to use camera sensors. Multi-speckle measurements have been demonstrated based on temporal speckle fluctuations using high speed linescan cameras,^12^ as well as based on spatial speckle contrast using standard CMOS imaging devices.^13^ While the linescan camera implementation preserves information about the full range of autocorrelation delays, the need for high-speed sampling and limited number of pixels available (hundreds to thousands) requires a heterodyne approach for coherent gain to overcome sensor noise.^14^^,^^15^ Sampling the spatial speckle contrast measured in an area some distance away from the illumination point provides high SNR potential as megapixel CMOS sensors are now available with low read noise and high frame rates. The latter approach, inspired by the laser speckle contrast imaging technique used for superficial perfusion imaging,^16^ has been termed speckle contrast optical spectroscopy/tomography (SCOS/SCOT)^13^^,^^17^ and has recently been demonstrated to allow light collection through multi-mode fiber bundles,^18^^,^^19^ and offer more than an order of magnitude improvement in SNR with a lower price for cerebral blood flow (CBF) monitoring versus DCS measurements at the same source–detector separation.^18^

Given the differences in how SCOS and DCS measurements derive blood flow from the fundamental scatterer motion, it is necessary to understand how both the sensitivity to cerebral perfusion, and the overall noise performance depend on system components and operating parameters for both methods. In this work, we seek to systematically explore SCOS and DCS performance envelopes and to provide guidance for investigators in the field planning studies involving optical monitoring of cerebral perfusion. Starting with Monte Carlo simulations of dynamic light transport in an MRI-derived, segmented head geometry, we add realistic measurement noise and consider operating scenarios constrained by representative hardware characteristics and light throughput. We then compare SCOS versus DCS intrinsic sensitivity and achievable contrast-to-noise ratio (CNR) for brain perfusion quantification.

The results provide a guide for choosing optimal illumination strategies, camera integration time (for SCOS), and source–detector separations. We conclude by discussing limitations of the study, including aspects of real-life measurements not considered in simulation and potential pitfalls. We made the code for generating the sensitivity, coefficient of variation (CoV), and contrast-to-noise ratio estimates from the outputs of Monte Carlo simulations run on an open-source dataset of MRI-derived head models publicly available as an example in the scatterBrains repository^20^ so that researchers can evaluate the performance of a particular DCS or SCOS system with the parameters different from what has been explored in this paper.

## 2 Methods

### 2.1 Diffuse Correlation Spectroscopy

DCS estimates the flow in tissue through the analysis of the normalized intensity autocorrelation function, $g_{2}(\tau )$. The autocorrelation of the intensity signal is related to the electric field temporal autocorrelation function $g_{1}(\tau )$ by the Siegert relation,^21^ expressed as

$$g_{2}(\tau )=1+\beta {\left|g_{1}(\tau )\right|}^{2},$$ (1)

where β is the coherence parameter,^22^ which is related to the coherence length of the source, the geometry of the measurement, number of modes detected, and the degree of environmental light contamination. The Siegert relation connects the measured signals to the underlying fluctuations of the electric field due to dynamic scattering events. The electric field autocorrelation function in the DCS measurement can be described as an integral of individual pathlength-specific correlation functions over the pathlength distribution detected. This form, given in Eq. (2),^23^ allows for the connection between the measured intensity autocorrelation function and the dynamics in the tissue:

$$g_{1}(\tau )=\int_{0}^{\infty }P(s)\exp(-\frac{1}{3}k_{0}^{2}n^{2}\langle \Delta r^{2}(\tau )\rangle \frac{s}{l^{*}})ds,$$ (2)

where P(s) is the distribution of pathlengths, s, taken by photons in the tissue, $k_{0}$ is the wavenumber of the detected light in a vacuum, n is the index of refraction of the sample, $\left\langle \Delta r^{2}(\tau )\right\rangle$ is the mean squared displacement of the scattering particles, and $l^{*}$ is the reduced, mean free path of photons in the tissue that is described as the inverse of the tissue’s reduced scattering coefficient $\left(l^{*}=\frac{1}{\mu_{s}^{\prime }}\right)$. For DCS measurements in tissue, the mean squared displacement term is assumed to reflect diffusive motion^24^ $\left(\langle \Delta r^{2}(\tau )\rangle =6BF_{i}\tau \right)$, where the blood flow index ($BF_{i}$) describes the effective diffusion coefficient. While this description of flow in vessels as a diffusive process is not immediately intuitive, multiple theoretical and simulation studies have examined the appropriateness of the model to describe the detected signals and found the diffusive process, arising from the shear-induced diffusion effect in dense colloids, such as blood, as a good description under standard DCS measurement conditions,^24^^–^^27^ though some conflicting theories have been proposed.^28^ When fitting correlation curves in this study, the model selected for $g_{1}(\tau )$ is that reflecting a semi-infinite sample measured in the reflectance geometry, given as^24^

$$g_{1}(\tau )=\frac{r_{b}\;\exp(-K(\tau )r_{1})-r_{1}\;\exp(-K(\tau )r_{b})}{r_{b}\;\exp(-\sqrt{3\mu_{a}\mu_{s}^{\prime }}r_{1})-r_{1}\;\exp(-\sqrt{3\mu_{a}\mu_{s}^{\prime }}r_{b})},$$ (3)

where $K(\tau )=\sqrt{3\mu_{a}\mu_{s}^{\prime }+6k_{0}^{2}n^{2}\mu_{s}^{\prime 2}BF_{i}\tau }$, $\mu_{a}$ is the optical absorption coefficient, $r_{1}=\sqrt{\rho^{2}+l^{*2}}$, ρ is the distance between the source and detector, $r_{b}=\sqrt{\rho^{2}+(l^{*}+2z_{b}{)}^{2}}$, $z_{b}=\frac{2}{3\mu_{s}^{\prime }}\frac{\left(1+R_{\mathrm{eff}}\right)}{\left(1-R_{\mathrm{eff}}\right)}$, and $R_{\mathrm{eff}}(n)=-1.440n^{-2}+0.71n^{-1}+0.668+0.0636n$.

### 2.2 Speckle Contrast Optical Spectroscopy

SCOS is a relatively newer technique that estimates the blood flow in tissue through the assessment of the measured speckle contrast of light collected after diffuse propagation in tissue.^13^ Detected light is projected to an imaging sensor, and the fluctuations in the speckle pattern over the exposure time of the sensor produce an image of the blurred speckle pattern. The degree of blurring is quantified by the speckle contrast of the image, K, which is defined as the ratio of the standard deviation of the intensity divided by the mean intensity $\left(K=\frac{\sigma_{I}}{\left\langle I\right\rangle }\right)$. In the absence of noise, the measured contrast is proportional to the DCS autocorrelation function though^29^

$$K_{f}^{2}=\frac{2\beta }{T_{\exp}}\int_{0}^{T_{\exp}}g_{1}^{2}(\tau )(1-\frac{\tau }{T_{\exp}})d\tau ,$$ (4)

where $K_{f}$ is the fundamental speckle contrast and $T_{\text{exp}}$ is the exposure time. In contrast to the single/few-mode fibers used in DCS measurements, light detection from the tissue in SCOS makes it possible to use large multimode fibers or multi-mode fiber bundles, greatly increasing the optical throughput, and, due to the number of pixels that are typically present in imaging sensors, allows for incredibly scalable, massively parallelized speckle channel detection.^18^^,^^19^

### 2.3 Simulation Models

#### 2.3.1 Description of segmented MRI models and Monte Carlo simulation

**Fig. 1:**
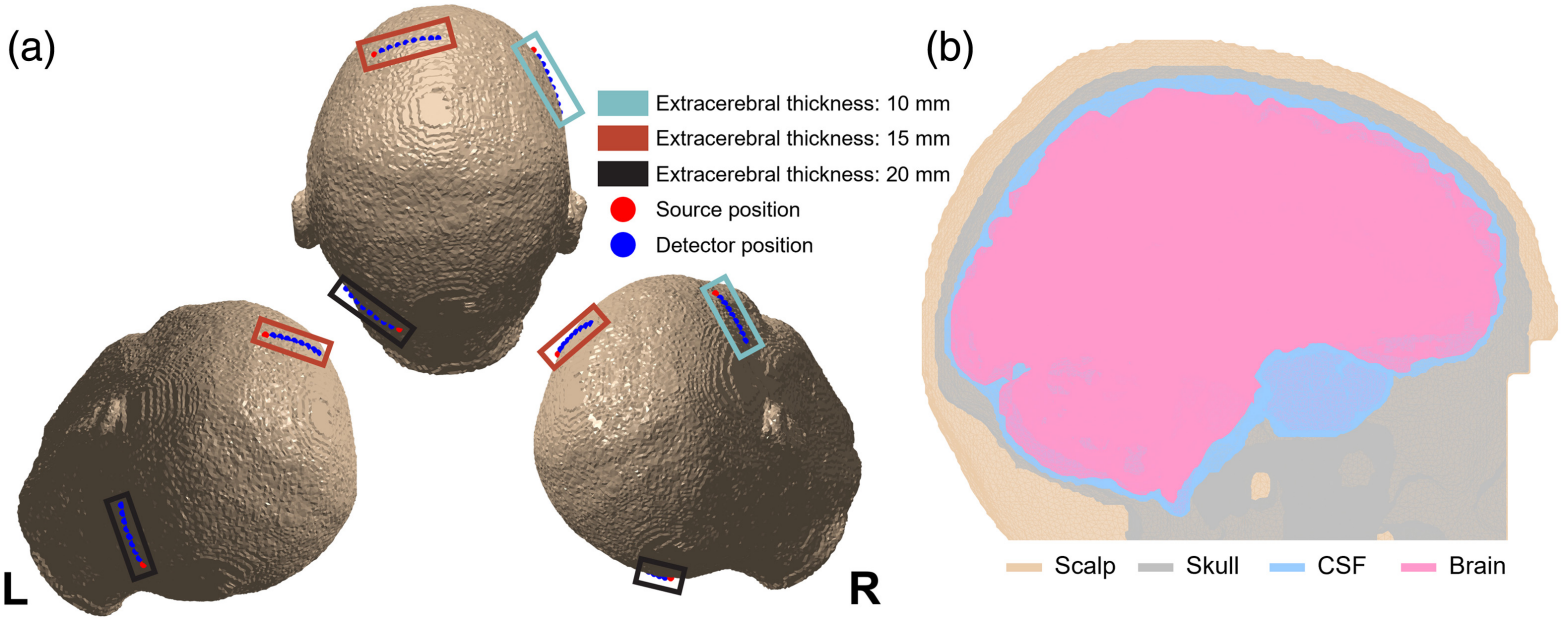
Depiction of the optical Monte Carlo geometry used for the simulations. The probe positions, shown in (a), were selected to achieve extracerebral thicknesses of 10, 15, and 20 mm. The tissue layers were segmented from the structural MRI scans and labeled as is shown in (b) in a sagittal plane near the midline. The gray matter and white matter tissue were combined into a single brain category.

This study utilized a structural MRI brain scan acquired as part of a larger study approved by Mass General Brigham Institutional Review Board.^30^^,^^31^ The tissue was segmented into four categories: scalp, skull, cerebrospinal fluid (CSF), and brain (combining gray and white matter) with optical and flow properties listed in Table 1. The segmented volume was converted to a mesh, with maximum targeted edge length of 5 mm and a maximum targeted tetrahedral volume of $200\;{\mathrm{mm}}^{3}$, as was described previously.^30^ We utilized a mesh-based model to represent the tissue, as mesh-based simulations have been shown to be more accurate when investigating curved structures and can provide efficient representation of both large- and small-scale features.^32^^,^^33^ Two brain flow states were considered, labeled “baseline” and “perturbed,” respectively, to allow for the assessment of the sensitivity to cerebral perfusion changes. To investigate the influence of extracerebral tissue on the measurements, three probe positionings were chosen in areas where anatomical features resulted in different distances from the surface of the scalp to the surface of the brain (10, 15, and 20 mm). The full details of the segmentation and probe placement are discussed in a previous work.^30^ The probe positions on the head [Fig. 1(a)] as well as an example of the segmented volume [Fig. 1(b)] are shown in Fig. 1.

**Table 1:** Optical properties at 850 and 1064 nm used for the Monte Carlo simulations. The bold characters indicate the changing value in the perturbed case.

Tissue	$\mu_{a}$ at 850 nm	$\mu_{s}^{\prime }$ at 850 nm	$\mu_{a}$ at 1064 nm	$\mu_{s}^{\prime }$ at 1064 nm	Baseline $BF_{i}$	Perturbed $BF_{i}$
Scalp	$0.164\;{\mathrm{cm}}^{-1}$	$7.4\;{\mathrm{cm}}^{-1}$	$0.11\;{\mathrm{cm}}^{-1}$	$5.3\;{\mathrm{cm}}^{-1}$	$1\times 10^{-8}\;{\mathrm{cm}}^{2}/s$	$1\times 10^{-8}\;{\mathrm{cm}}^{2}/s$
Skull	$0.155\;{\mathrm{cm}}^{-1}$	$8.1\;{\mathrm{cm}}^{-1}$	$0.13\;{\mathrm{cm}}^{-1}$	$5.8\;{\mathrm{cm}}^{-1}$	$1\times 10^{-10}\;{\mathrm{cm}}^{2}/s$	$1\times 10^{-10}\;{\mathrm{cm}}^{2}/s$
CSF	$0.017\;{\mathrm{cm}}^{-1}$	$0.1\;{\mathrm{cm}}^{-1}$	$0.122\;{\mathrm{cm}}^{-1}$	$0.07\;{\mathrm{cm}}^{-1}$	$1\times 10^{-10}\;{\mathrm{cm}}^{2}/s$	$1\times 10^{-10}\;{\mathrm{cm}}^{2}/s$
Brain	$0.170\;{\mathrm{cm}}^{-1}$	$11.6\;{\mathrm{cm}}^{-1}$	$0.17\;{\mathrm{cm}}^{-1}$	$8.3\;{\mathrm{cm}}^{-1}$	$6\times 10^{-8}\;{\mathrm{cm}}^{2}/s$	$7.2\times 10^{-8\;{\mathrm{cm}}^{2}/s}$

Optical Monte Carlo simulations were run using MMC^32^ with $1.5\times 10^{9}\;\text{photon}$ trajectories launched per simulation. Source–detector separations between 5 and 40 mm were simulated at a spacing of 5 mm. For each source–detector separation, the unnormalized, electric field autocorrelation function $\left(G_{1}(\tau )\right)$ was calculated using the discretized form of Eq. (2), given as^24^

$$G_{1}(\tau )=\frac{1}{N_{p}}\overset{N_{p}}{\underset{n=1}{\sum }}\;\exp(-\frac{1}{3}k_{0}^{2}\overset{N_{t}}{\underset{i=1}{\sum }}Y_{n,i}{\left\langle \Delta r^{2}(\tau )\right\rangle }_{i})\exp(-\overset{N_{t}}{\underset{i=1}{\sum }}\mu_{a,i}L_{n,i}),$$ (5)

where $N_{p}$ is the number of detected photons, $N_{t}$ is the number of tissue categories, $Y_{n,i}$ is the dimensionless momentum transfer of photon n occurring in tissue category i, and $L_{n,i}$ is the partial pathlength of photon n in tissue category i. For this work, two implementations of DCS measurements and one SCOS implementation were evaluated. For DCS, the first was DCS operating at 850 nm based on silicon SPADs and the second was DCS operating at 1064 nm based on super-conducting nanowire detectors (SNSPDs).^34^ For SCOS, the implementation model was based on 850 nm operation with a CMOS camera with pixel number, read noise, and quantum efficiency properties based on aggregated camera properties for commercially available cameras.^35^ Although great success has recently been shown in the application of interferometric methods to both SCOS and DCS,^14^^,^^15^^,^^36^^–^^41^ we restricted the analysis in this work to strictly homodyne methods. The photon flux at each simulated source–detector separation was estimated from the diffuse reflectance output from MMC and scaling the results based on estimates from previous studies.^42^ For 850 and 1064 nm, the photon flux per mode per ANSI limited source at a 25 mm source–detector separation was estimated to be 10 and 67.1 kcps, respectively.^42^

#### 2.3.2 Noise model used for DCS simulations

To evaluate the simulated performance of DCS measurements, we utilized the correlation noise model,^43^^,^^44^ given as

$$\sigma (\tau )=\sqrt{\frac{T}{t}}[\begin{array}{c} \beta^{2}\frac{(1+e^{-2\Gamma T})(1+e^{-2\Gamma \tau })+2\frac{\tau }{T}(1-e^{-2\Gamma T})e^{-2\Gamma \tau }}{1-e^{-2\Gamma T}}+..\\ 2{\left\langle n\right\rangle }^{-1}\beta (1+e^{-2\Gamma \tau })+..\\ {\left\langle n\right\rangle }^{-2}(1+\beta e^{-\Gamma \tau }) \end{array}{]}^{\frac{1}{2}},$$ (6)

where σ(τ) is the standard deviation of the correlation function at a time lag τ, T is the width of the correlation function time bin, t is the averaging time of the measurement, ⟨n⟩ is the average count rate per correlation time bin T, and Γ is the decay rate of the temporal intensity autocorrelation if it were modeled as a single exponential decay, i.e., $g_{2}(\tau )=1+\beta \;\exp(-\Gamma \tau )$. To determine the noise performance of DCS for a given measurement arrangement, 100 realizations of Gaussian white noise of amplitude σ(τ) for all delays, τ, were added to the Monte Carlo simulation generated $g_{2}(\tau )$ curve and the 100 noisy curves were fit for $BF_{i}$ values using the semi-infinite solution to the correlation diffusion equation with estimated optical properties (850 nm: $\mu_{a}=0.15\;{\mathrm{cm}}^{-1}$, $\mu_{s}^{\prime }=8.5\;{\mathrm{cm}}^{-1}$; 1064 nm: $\mu_{a}=0.15\;{\mathrm{cm}}^{-1}$, $\mu_{s}^{\prime }=6.2\;{\mathrm{cm}}^{-1}$) with the assumption that the coherence parameter, β, was known.

#### 2.3.3 Calculation of speckle contrast and speckle contrast noise

The fundamental squared speckle contrast ($K_{f}^{2}$) was computed for each measurement condition and source–detector separation based on Eq. (4). To evaluate the simulated performance of SCOS measurements, we utilized the recently published model for speckle contrast noise.^45^ The measured speckle contrast not only exclusively reflects the fundamental speckle contrast ($K_{f}$) due to the tissue dynamics but also reflects contributions from shot noise ($K_{s}$), read noise ($K_{r}$), and dark noise ($K_{d}$). Thus, the square of the measured speckle contrast (K) can be expressed as a sum of the squares of each contrast term [Eq. (7)].

$$K^{2}=K_{f}^{2}+K_{s}^{2}+K_{r}^{2}+K_{d}^{2}.$$ (7)

In this work, as was done previously,^45^ due to the range of exposure times investigated, we neglected the contribution from dark noise. While this assumption breaks down in the long exposure regime (10s of ms, Sec. S1 in the Supplemental Material), measurement systems do not typically operate in this regime. To estimate the CoV of the blood flow index from the simulated speckle contrast, noise estimates ($\sigma_{K^{2}}$) for each of the contrast terms were computed as described previously using a dynamic speckle evolution model^45^ for each measurement condition and source–detector separation.^45^ 100 noisy realizations were sampled from a normal distribution with mean $K_{f}^{2}$ and standard deviation $\sigma_{K^{2}}$. The data were fit for $BF_{i}$ using an inverse model based on Eq. (4) and the solution to the correlation diffusion equation based on the semi-infinite geometry for $g_{1}(\tau )$ with the same optical properties used for DCS fitting and the assumption that the coherence parameter, β, is known. Fitting is performed by reducing the error between the fundamental squared contrast of the measurement $\left(K_{f}^{2}\right)$ and the modeled contrast computed as a function of the exposure time of the measurement and the estimated blood flow index. In these simulations, the contributions to the squared speckle contrast from shot noise ($K_{s}^{2}$) and read noise ($K_{r}^{2}$) were assumed to be accurately removed, though in reality, the inaccurate subtraction of these terms may lead to drift in the measured blood flow.^18^^,^^45^

#### 2.3.4 Description of comparison metrics

To estimate the sensitivity of each simulated measurement to changes in the CBF signal, two sets of simulations were run: a baseline flow state and a brain activation flow state (+20% CBF, Sec. S2 in the Supplemental Material). The fraction of the flow change recovered was used as a metric for the sensitivity of the measurement to the CBF signal, given as

$$\text{Sensitivity}=\frac{\left(\frac{\Delta BF_{i}}{BF_{i,\text{baseline}}}\right)}{\left(\frac{{\Delta \mathrm{CBF}}_{i}}{{\mathrm{CBF}}_{i,\text{baseline}}}\right)}.$$ (8)

The degree of cerebral sensitivity necessary to make an effective measurement depends on the measurement conditions and methods used to address the extracerebral contamination, including use of short separation regression,^46^ use of overlapping channels,^47^ use of repeated stimuli for block averaging, multi-layer or Monte Carlo fitting,^31^^,^^48^ or the use of the pressure modulation technique.^49^ For evaluation of the results, we estimate a cerebral sensitivity of ≥10% is acceptable for effective measurements given the requirements of CoV and CNR described below. The noise performance of the measurement was assessed by computing the CoVof the fit $BF_{i}$ values, given as

$$\mathrm{CoV}=\frac{\sigma_{BF_{i}}}{\mu_{BF_{i}}},$$ (9)

where $\sigma_{BF_{i}}$ is the standard deviation of the fit results and $\mu_{BF_{i}}$ is the average value of the fit results. Variability in blood flow due to typical physiological processes (cardiac, respiratory, Meyer waves, etc.^50^) has been estimated to contribute to a CoV of ∼0.1 for measurements of CBF.^51^ Instrument based CoV computed from the simulations is deemed acceptable if the estimated CoV is less than the physiologic noise (CoV≤0.1). To combine both the estimates of sensitivity and CoV, we computed a contrast-to-noise metric, which is the ratio of the sensitivity to the CBF and the CoV, given as

$$\mathrm{CNR}=\frac{\text{Sensitivity}}{\mathrm{CoV}}.$$ (10)

For an effective measurement, we desire a CNR≥1. A graphic describing the full simulation pipeline can be found in Sec. S4 in the Supplemental Material.

#### 2.3.5 Description of the characteristics of the simulated hardware

The descriptions of the relevant parameters for the three simulated detection systems (850 nm DCS, 1064 nm DCS, and 850 nm SCOS) and the two simulated laser sources (850 and 1064 nm) are given in Tables 2 and 3, respectively. Each of the DCS systems is based on a single-element detector module, and the SCOS system is based on a single CMOS camera with typical noise performance. The hardware parameters selected for this work were based on hardware components used by our group previously and do not constitute an exhaustive list of possible configurations, though they are still representative of the general landscape of possible configurations. In addition to the different hardware parameters, different conditions of light collection from the tissue were also explored. For the DCS systems, we evaluated the performance differences between single-mode fiber (780 HP, two polarization modes) and few-mode fiber (SMF28, 12 total modes at 850 nm, six total modes at 1064 nm) as several works have utilized few-mode fiber, especially for measurements made at longer source–detector separations.^5^^,^^52^^,^^53^ To more effectively model the influence of dark counts on the DCS signal, using a previously developed state space model of a single photon detector,^54^ realistic series of photon count timestamps were generated considering the fluctuating speckle signal intensity, randomly occurring dark counts and the detector hold off time. The $BF_{i}$ was fit from the resulting autocorrelation functions of the generated timestamp series. We explored a similar concept with the SCOS simulations with the projection of light onto the sensor. By altering the ratio of the size of the speckle projected onto the camera to the pixel size, known as the s/p ratio, the number of independent fiber modes projected onto a single pixel would be altered, as was explored in previous work.^45^ For the SCOS system, the simulated light was collected from the tissue by a fiber bundle consisting of 2000, 50  μm fibers with a numerical aperture (NA) of 0.66. This bundle specification gives an estimated number of fiber modes equal to $1.48\times 10^{7}$ at 850 nm. For a uniformly illuminated camera sensor, the square root of the ratio of number of pixels (2.5 megapixels) and the number of fiber modes sets the minimum s/p ratio achievable. For this simulation, the minimum s/p ratio is equal to 0.41, as further decreases in s/p ratio would result in underfilling the camera and reducing the number of pixels used for speckle contrast calculation.

**Table 2:** Description of simulated detection hardware.

	DCS @ 850 nm (Si SPAD^55^)	DCS @ 1064 nm (SNSPD^56^)	SCOS @ 850 nm^35^
Detector QE	55%	90%	20%
Dark current/count rate	0, 500, 1000, 1500 cps	0 cps	$0\;e^{-}/s$
Read noise	0	0	$2.5\;e^{-}$
Max frame rate	Free running^a^	Free running^a^	150 Hz
Number of pixels	1	1	2.5 megapixels

a While the single photon detectors are free running, they both exhibit hold off times after a photon detection event, which limits the total possible number of photons counted per second. Si-SPAD: 22 ns ($4.5\times 10^{7}\;\mathrm{cps}$ saturation limit), SNSPD: 33 ns ($3.0\times 10^{7}\;\mathrm{cps}$ saturation limit).

**Table 3:** Description of the simulated laser hardware.

	Laser source @ 850 nm^57^	Laser source @ 1064 nm^58^
ANSI standard^59^ limited power for a 3.5 mm diameter spot	38 mW	100 mW
Max laser output power	300 mW (7.9× ANSI single spot)	1 W (10× ANSI single spot)
Illumination strategies enabled	Up to 8 source points (DCS); 300 mW illumination with 1/7.9 duty cycle (SCOS)	Up to 10 source points (DCS)

#### 2.3.6 Description of performance comparisons made across different measurement settings

For DCS and SCOS, there are several comparable measurement conditions that can be adjusted to tune either the sensitivity of the measurement to the cerebral signal or the SNR of the measurement. Namely, we find parallels between the range of the correlation function decay that is fit for the $BF_{i}$ and the exposure time used for the camera sensor; the strategy of source light projection to the tissue that maximizes the number of photons per mode given the duty cycle of the measurement; and the number of modes projected to the single photon detector and the s/p ratio used for the camera. For each comparison, we quantified the sensitivity to the cerebral signal, the CoV of the measurement at a 10 Hz sample rate, and the contrast-to-noise ratio of the measurement at 10 Hz. We chose this sampling rate to meet the requirements of functional measurements, where the cardiac pulsatile signal must be filtered out without aliasing, as well as fast enough to sample physiological dynamics in clinical monitoring. As it is not feasible to explore the full matrix of combinations of these parameters, we instead focused on each one in turn, making simplifying assumptions about the other three. In Sec. 3.1, we evaluated the performance impact of different fitting ranges for the DCS correlation function and exposure times for SCOS acquisition. In Sec. 3.2, we utilized the optimal fitting range and exposure time found in Sec. 3.1 and evaluated the optimal source delivery strategy for both DCS and SCOS. In Sec. 3.3, taking the optimal fitting range/exposure time and laser source delivery strategy, we evaluated the influence of the number of independent fiber modes sampled by a single detector by modifying the simulated fiber used in the case of DCS and the s/p ratio for SCOS. And finally in Sec. 3.4, we evaluated the performance of DCS and SCOS $BF_{i}$ measurements at the optimized operating condition as explored in Secs. 3.1–3.3 as a function of extracerebral tissue thickness. The simplifying assumptions made for each section are detailed in Table 4, with the shaded diagonal representing the parameter being explored in each section.

**Table 4:** Description of the simplifying assumptions for each results section.

	DCS fit range/SCOS exposure time	Illumination strategy	Number of modes per detector	Extracerebral tissue thickness
Section 3.1	DCS: explored for values from 10% to 100% of the decay range SCOS: Exposure times between 1 μs and 100 ms explored at all source–detector separations	DCS and SCOS: CW illumination with a single ANSI limited source	DCS: unpolarized single-mode detection fiber (two modes) SCOS: unpolarized detection with s/p ratio equal to 1	DCS and SCOS: 15 mm extracerebral thickness
Section 3.2	DCS: 100% of $g_{2}(\tau )$ decay is used SCOS: Exposure times between 1 μs and 100 ms explored at short (15 mm) and long (30 mm) source–detector separations	DCS: three strategies explored (1) Single CW source (2) Multiple CW sources (3) Single pulse width modulated source SCOS: four strategies explored, (1) Single CW source (2) Multiple CW sources (3) Single pulse width modulated source where the max laser power is modified to satisfy $P_{\text{in}}*f_{s}*T_{\exp}\leq P_{\mathrm{ANSI}}$, (4) Single pulse width modulated source where the frame rate of the camera is modified to satisfy $P_{\max}*f_{s}*T_{\exp}\leq P_{\mathrm{ANSI}}$	DCS: unpolarized single-mode detection fiber (two modes) SCOS: unpolarized detection with s/p ratio equal to 1	DCS and SCOS: 15 mm extracerebral thickness
Section 3.3	DCS: 100% of $g_{2}(\tau )$ decay is used SCOS: exposure times between 1 μs and 100 ms explored for short (15 mm) and long (30 mm) source detector separations as well as a comparison between all source–detector separations at the exposure time, which results in optimal CNR	DCS: CW illumination with a single ANSI limited source SCOS: the pulse width modulated illumination strategy (#3 or #4 explored in Sec. 3.2) which maximized CNR	DCS: unpolarized detection with a single-mode fiber and a few-mode fiber SCOS: unpolarized detection with s/p ratios ranging from 2 to the minimum s/p ratio (0.41)	DCS and SCOS: 15 mm extracerebral thickness
Section 3.4	DCS: 100% of $g_{2}(\tau )$ decay is used SCOS: The exposure time, which results in optimal CNR is selected for each simulated condition	DCS: either CW illumination with a single ANSI limited source or pulse width modulated illumination, whichever approach maximized CNR SCOS: The pulse width modulated illumination strategy (#3 or #4 explored in Sec. 3.2), which maximized CNR	DCS: unpolarized single-mode detection fiber (two modes) SCOS: minimum s/p ratio (0.41) allowed by the number of simulated modes and number of pixels	DCS and SCOS: three thicknesses explored from three different positions on the head

## 3 Results

### 3.1 Effect of Correlation Function Fitting Range and Exposure Time on Expected Performance of DCS and SCOS

**Fig. 2:**
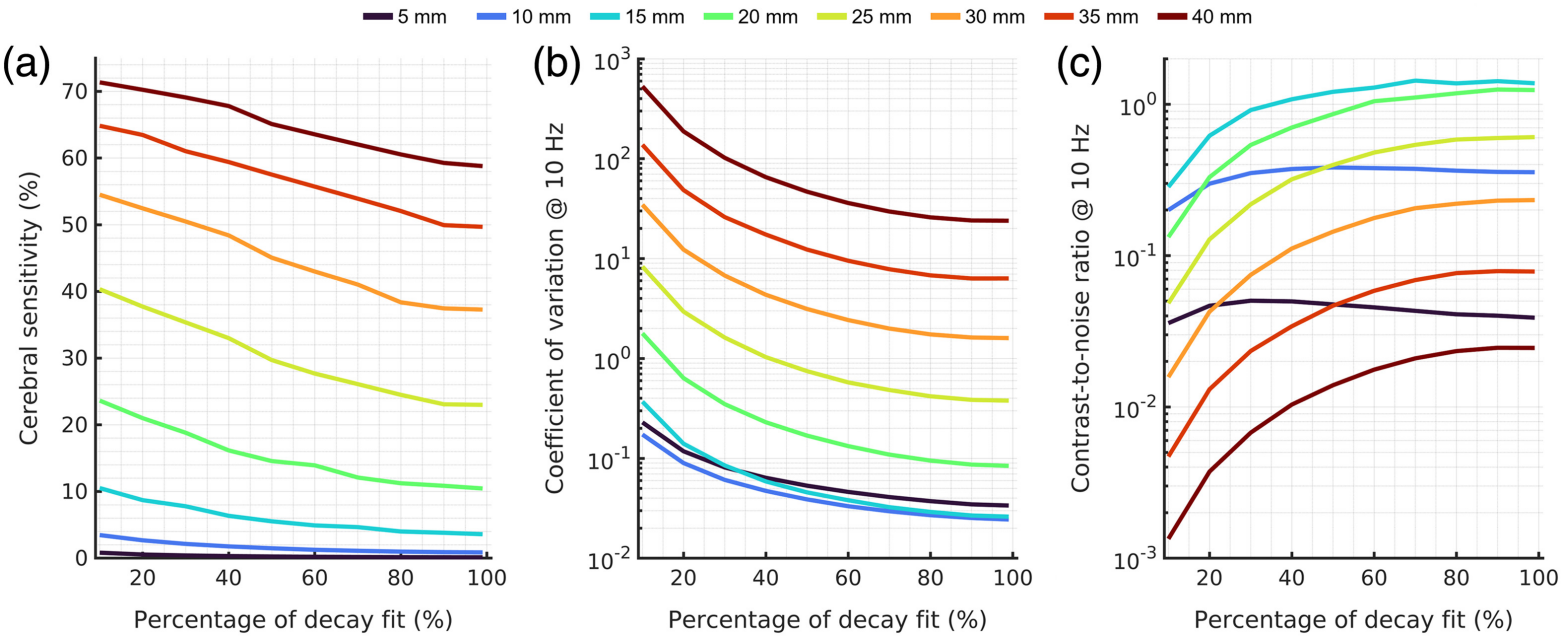
Comparison of the performance of DCS at 850 nm as a function of the percent of the decay of the autocorrelation function that is used for $BF_{i}$ fitting. (a) The sensitivity to the cerebral signal has a monotonically increasing relationship with source–detector separation, and a monotonically decreasing relationship with the percentage of the curve that is fit. (b) The CoV monotonically decreases when more of the correlation function is fit. Dividing the sensitivity by the CoV results presented in (b), the contrast-to-noise ratio for each source–detector separation can be seen in (c). Two relatively distinct patterns can be seen when comparing short separation measurements (SDS≤10  mm) to the rest of the source–detector separations. The reduction in the CoV when the fitting range of the curve is increased is less at shorter separations, which increases the relative weight of the decrease in cerebral sensitivity as the fitting range of the curve is increased. For longer separation measurements, the decrease in CoV is much greater as the fitting range is increased and leads to optimal measurements being made when the entire curve is considered for the fit.

As has been evaluated previously,^60^ fitting the earlier part of the correlation function increases the sensitivity of the measurement to the faster flows measured and to the cerebral signal, but at the cost of the signal to noise ratio of the fit. In Fig. 2, the results for sensitivity [Fig. 2(a)], CoV [Fig. 2(b)], and contrast-to-noise ratio [Fig. 2(c)] for each source–detector separation for the 15 mm extracerebral thickness geometry are shown for 850 nm DCS with CW laser illumination with a single ANSI limited source and single-mode fiber detection. For the long separation measurements (SDS≥20  mm), the CNR of the measurement is maximized when the entire curve is fit, with the decrease in the CoV outweighing the reduced cerebral sensitivity. While measurements at short separation are not typically analyzed for their brain sensitivity, higher contrast-to-noise for the cerebral signal is found when fitting the early part of the curve, showing the relatively small contributions of long path photons to the overall measurement at short separation.^61^ This effect at short separation is relatively small, as shown in Fig. 2(c), and thus for the remaining DCS results presented in this work, the fitting range of the correlation function was set to be the entire curve. For DCS at 850 nm with a single SPAD detector, maximal CNR to the cerebral signal is achieved at 15 mm in an area where the brain is 15 mm under the scalp.

**Fig. 3:**
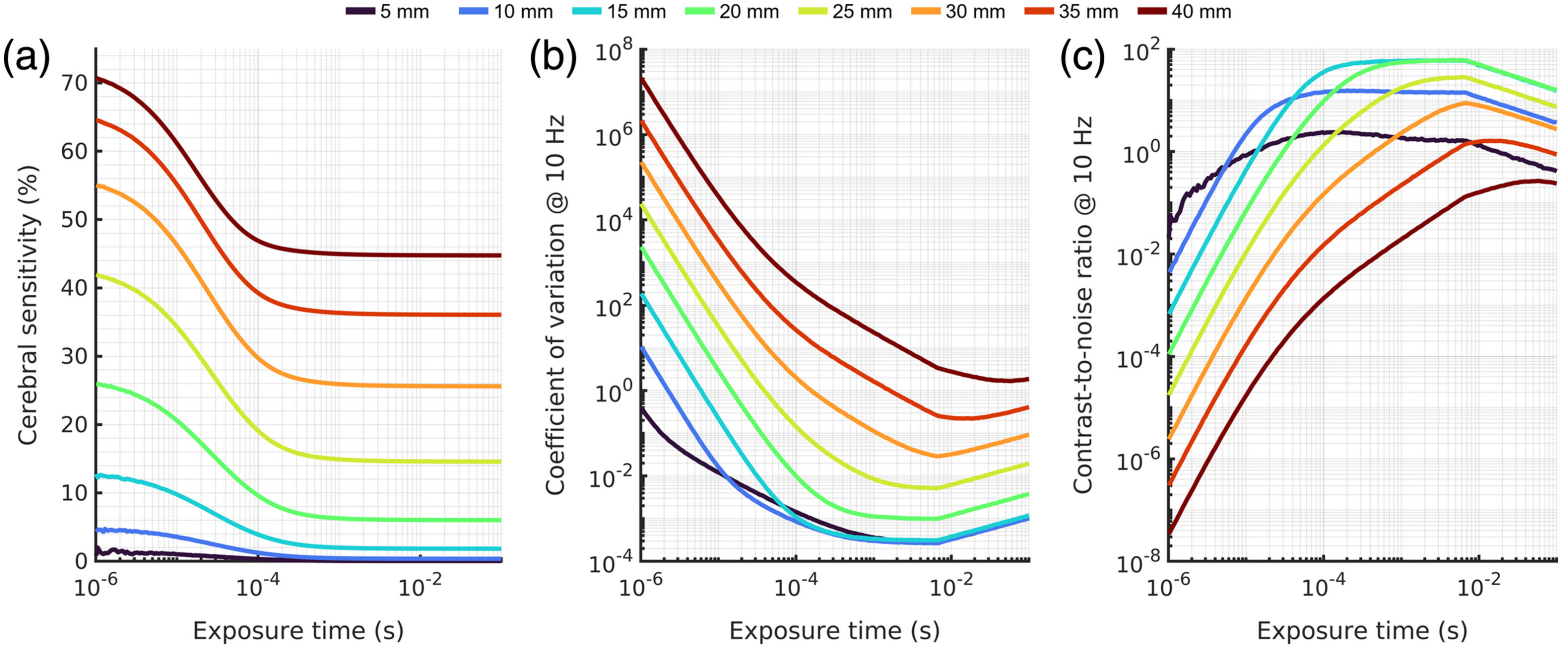
Comparison of SCOS performance as a function of exposure time. (a) Cerebral sensitivity for SCOS matches DCS sensitivity when extremely short exposure times are used. For exposures longer than ∼300  μs, for all source–detector separations explored, sensitivity drops to a steady state value that is ∼10% to 60% of the maximal value. The reduced sensitivity is compensated for by the improved CoV of the fit blood flow index (b), which reflects the benefits of massively parallelized speckle detection. Combining the two metrics, (c) the contrast-to-noise ratio plots show a relatively complex relationship with exposure time, reflecting the interplay of increasing exposure time with frame averaging and the effect of the noise sources.

In Fig. 3, we show the expected SCOS performance in the same simulation geometry as a function of the exposure time of the camera. For these results, the simulations reflect a spatial sampling of the speckle where the pixel size matches the speckle size (s/p ratio=1) and are performed with a single ANSI limited source emitting continuously, which does not take full advantage of the reduced duty cycle of the camera at shorter exposure times. These simulated conditions match the initial implementations of SCOS.^13^ Despite the suboptimal conditions for SCOS measurements, a ∼40× improvement in maximal CNR is achieved as compared to the maximal CNR achieved by single channel DCS at 850 nm. Similar trends are observed when comparing SCOS performance to DCS performance, with cerebral sensitivity decreasing with increasing exposure time, though the CoV changes observed differ slightly at the limit of longer exposures. For DCS, which measures relatively continuously, except during the hold-off times of the single photon detectors, the relationship of the noise of the measurement to the sampling rate of the measurement is relatively straight forward and is scaled as the square root of the sampling rate. For SCOS, the maximum frame rate of the camera and the exposure time of the camera interplay with the final sampling rate of the SCOS $BF_{i}$ measurement, and the degree of frame averaging at a particular sampling rate is affected. As shown in Fig. 3(b), the CoV has roughly two distinct regimes; the first where the exposure time does not limit frame rate (which is instead limited by the maximal frame rate of the camera hardware (150 Hz, 6.67 ms)) and increasing the exposure time increases the number of detected photons without affecting frame averaging, and the second where the longer exposure time requires a slower frame rate, thereby reducing frame averaging. For measurements at short source–detector separations, the contrast-to-noise ratio increases to an asymptotic value before reaching the critical exposure time, then decays with increasing exposure time as frame averaging is reduced. For measurements made at long source–detector separations, due to the reduced photon flux, the plateau is not reached before the critical exposure time. For these source–detector separations, performance can be improved by extending the exposure time beyond the critical exposure time, and up to a certain increase in exposure time, the benefit of collecting more light with a longer exposure outweighs the decrease in frame averaging.

### 3.2 Effect of Light Source Delivery Strategy on DCS and SCOS

As noted previously, the use of a continuous wave laser emitting continuously causes some light to be “wasted” during the unexposed time of the camera sensor. This effect is less prevalent in the DCS measurements, as the duty cycle for a single photon detector-based system is ∼100% for the conditions in which DCS measurements are typically made but can become substantial for SCOS, especially at shorter exposure times where the duty cycle is inherently low. Laser sources typically emit more power than what is allowed by ANSI skin exposure limits, requiring attenuation. To take advantage of this excess laser power, the input can be pulse width modulated to keep the average power at the ANSI limit while utilizing the full power of the laser during the detector-on period.^18^ Alternatively, multiple source positions^14^ or larger spot sizes^9^^,^^15^ can be used to increase the number of photons available for detection while remaining in compliance with the ANSI limit of power delivery per unit area. Four laser delivery strategies were explored in this work: (1) continuous illumination with a single ANSI limited source with a diameter of 3.5 mm (same as in Figs. 2 and 3). (2) Continuous illumination with multiple ANSI limited sources arrayed in non-overlapping fashion around the detector, limited in number by the maximum output of the laser, to deliver the full power of the laser with continuous illumination. (3) A pulsed laser strategy where the laser input power ($P_{\text{in}}$) is modulated such that $P_{\text{in}}*f_{s}*T_{\exp}\leq P_{\mathrm{ANSI}}$ and the frame rate (fs) is not adjusted. (4) A pulsed laser strategy where the frame rate of the camera is modulated such that $P_{\max}*f_{s}*T_{\exp}\leq P_{\mathrm{ANSI}}$ and the max laser power is used.

**Fig. 4:**
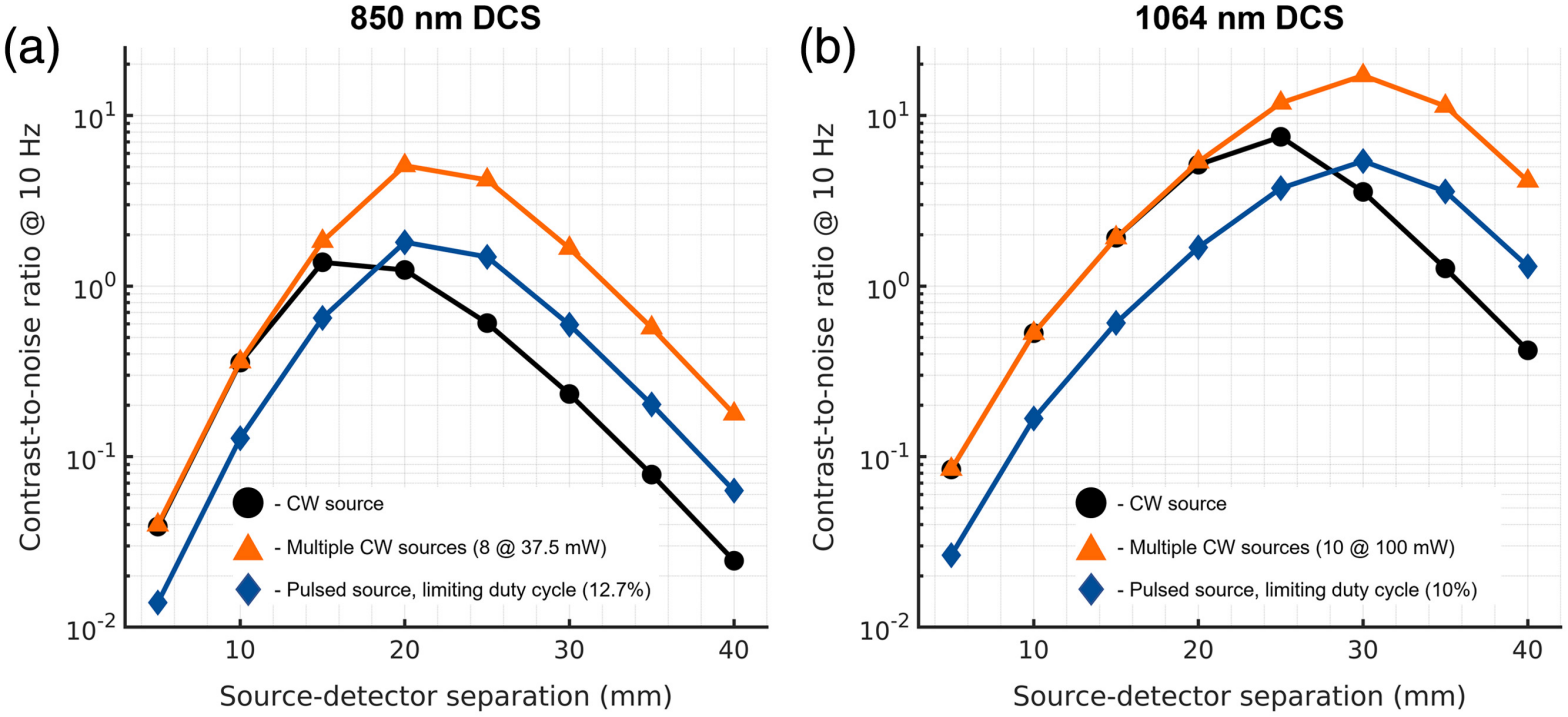
Comparison of the simulated CNR of DCS measurements at (a) 850 and (b) 1064 nm as a function of source–detector separation and laser input strategy. For both 850 nm DCS and 1064 nm DCS, for source–detector separations with sufficiently high count-rate with a single CW source, the use of a pulsed laser source with a reduced duty cycle is seen to limit the observed CNR. This relationship flips with longer source–detector separations as the count rate drops and the use of a pulsed laser with a reduced duty cycle is seen to improve the CNR.

For DCS, we compared the performance of the strategies for both 850 nm DCS [Fig. 4(a)] and for 1064 nm DCS [Fig. 4(b)]. Because the duty cycle of a DCS measurement is ∼100%, for the comparison of pulsed laser strategies, we evaluated a single pulsed laser strategy where the duty cycle of the measurement is chosen to be the ratio of the max laser power divided by the ANSI limit for a single spot with a 3.5 mm diameter (Table 3). This is equivalent to the frame rate limiting approach given as illumination strategy 4 for SCOS. The use of the pulsed laser strategy in DCS has both advantages and disadvantages relative to the single source CW illumination approach. For short-separation measurements that exhibit high count rates (>100  kcps), the predicted noise of the correlation function, given in Eq. (6), will depend much more on (1) the first term within the square root, which has no dependence on count rate, and (2) the averaging time of the measurement. The predicted consequence of this dependence is observed in the short separation measurements, where the simulated CNR for the pulsed laser strategy is reduced by a factor of $∼\sqrt{\text{Duty Cycle}}$. For longer source–detector separation measurements, the relationship is flipped, with the increase in the instantaneous count rate outweighing the reduced averaging, and the improvement in CNR by a factor of $∼\sqrt{1/\text{Duty Cycle}}$. For the multi-source CW strategy, in the photon starved, long source–detector separation regime, the improvement in CNR over the single-source CW strategy is approximately the ratio of the maximum input power to the ANSI-limited single source input power. As has been previously reported,^34^^,^^42^ we observe in these simulations that DCS at 1064 nm outperforms DCS at 850 nm in terms of the contrast-to-noise ratio of the cerebral signal.

**Fig. 5:**
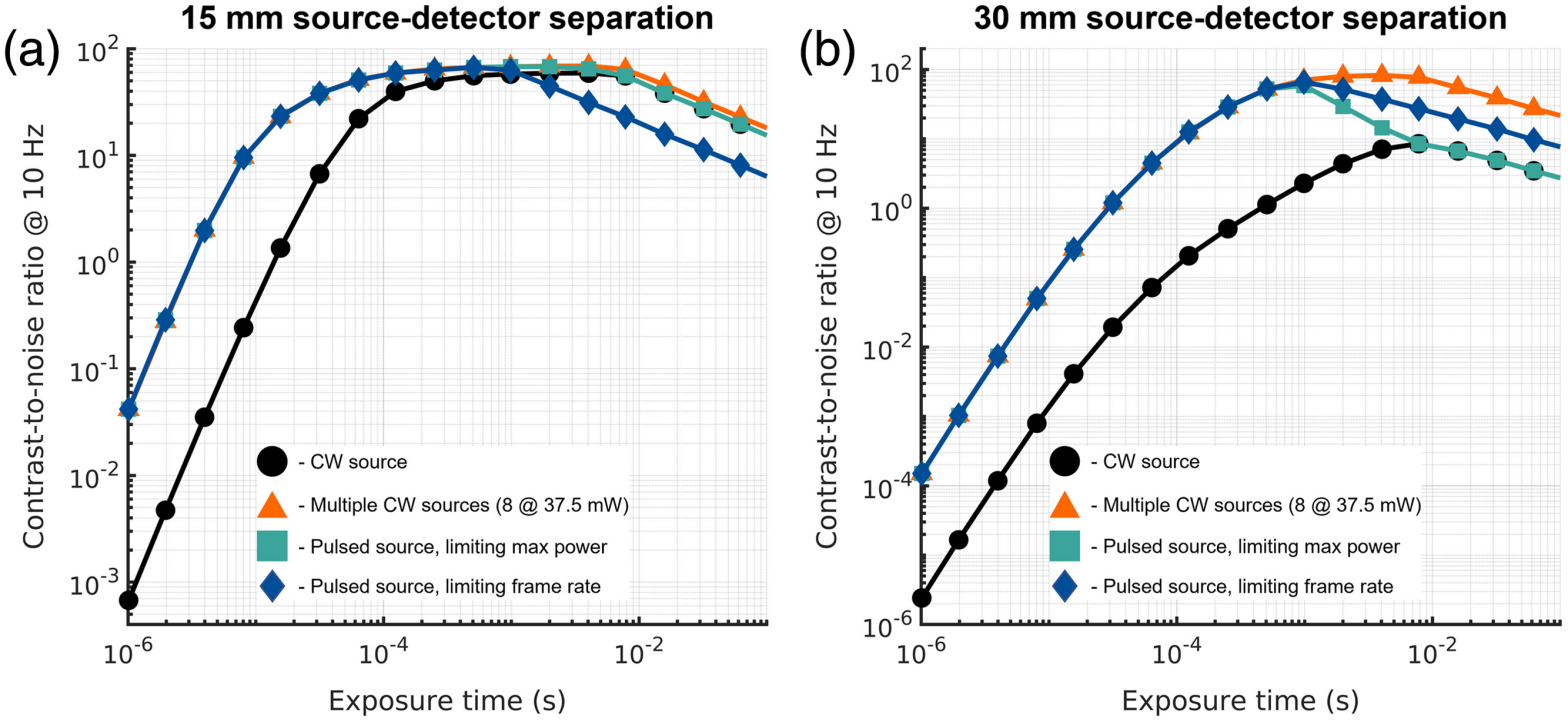
Comparison of source illumination strategies on the CNR of the SCOS measurement for (a) a short separation measurement and (b) a long separation measurement. For both sets of simulated measurements, the multiple CW source strategy sets the performance ceiling for the pulsed source strategies, though the strategy that is advantageous at each source–detector separation is different. For the short separation, limiting the source power in favor of averaging is advantageous, whereas at the long separation, the opposite is true.

A comparison of the effect that each of these strategies has on the CNR of SCOS measurements as a function of exposure time is shown in Fig. 5. In the case of the short separation measurement [15 mm, Fig. 5(a)], because shot-noise limited performance is reached at shorter integration times, by applying the frame rate limiting pulsed strategy, the reduction in frame averaging is an overall stronger influence on the SNR of the measurement, and an overall decrease in CNR is observed. In the case of the long separation measurement [30 mm, Fig. 5(b)], shot-noise limited performance is not reached before the exposure time begins limiting the frame rate, so a different behavior is observed, with distinct separation between the performance of the multi-source and pulsed source strategies. A more in-depth discussion of the shapes of the CNR curves can be found in Sec. S3, Fig. S3 in the Supplemental Material. For both sets of simulated measurements, the multiple CW source strategy provides the most consistently improved results, as it provides the max power of the laser continuously with the full duty cycle of a CW measurement, though nearly equivalent performance can be achieved through one of the pulsing strategies for both source–detector distances. These observations parallel the DCS results presented in Figs. 4(a) and 4(b). For long separation measurements, best sensitive to cerebral physiology, these results suggest that if there is enough room for probe placement on the head, a multi-source strategy provides the best CNR for DCS and SCOS measurements, though care must be made to ensure that the spread of the total photon pathlength distribution across all source locations remains within the coherence length of the laser to avoid reducing the coherence parameter of the measurement.

### 3.3 Comparison of the Effect of Increased Modal Content on DCS and SCOS Signal Quality

**Fig. 6:**
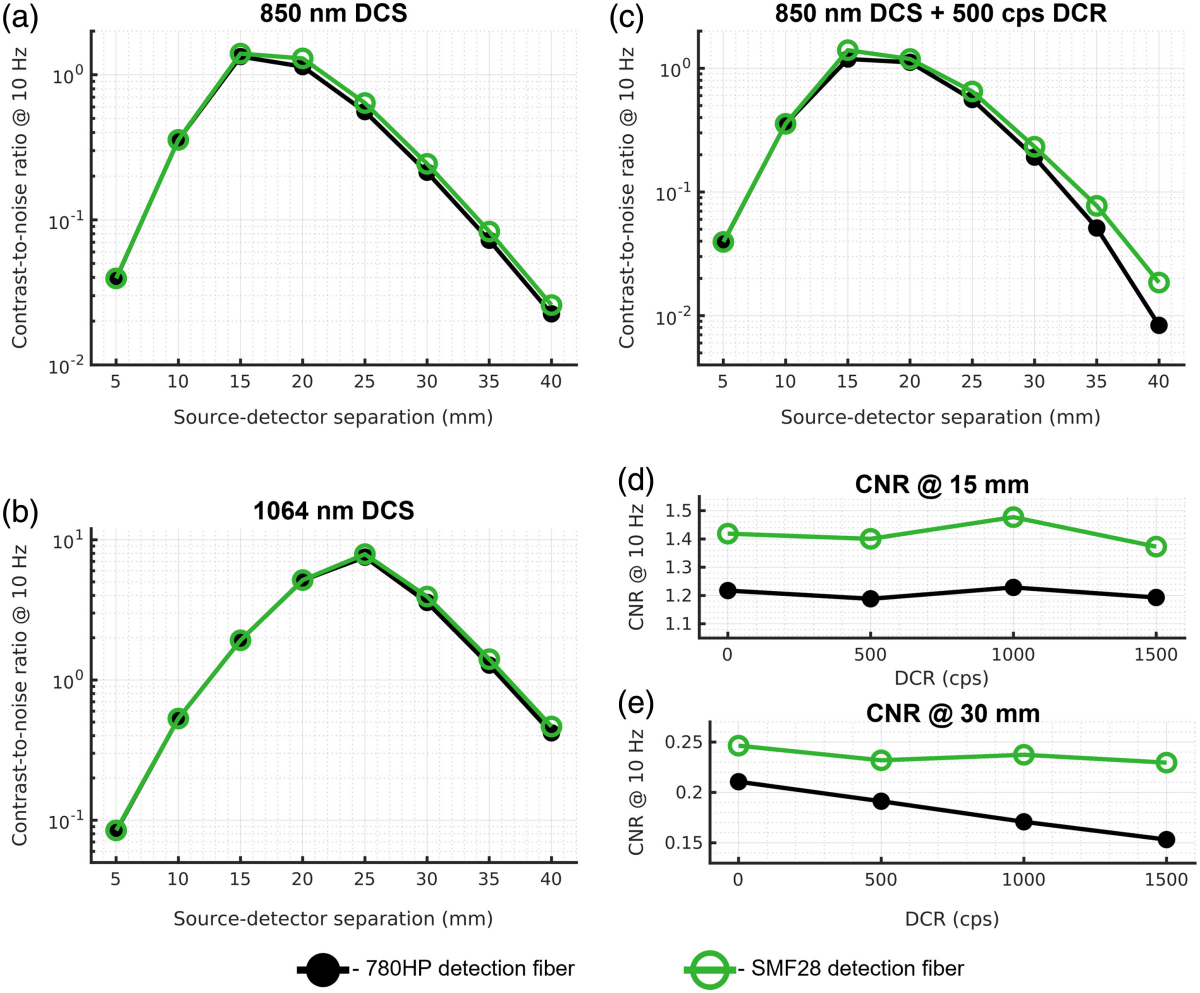
Comparison of DCS contrast-to-noise ratio when fibers with different number of guided modes are used. For noiseless detectors, shown in (a) and (b), the use of few-mode fiber produces a modest improvement in performance, maximized at longer source detector separations. The influence of dark noise on the CNR is explored in (c), where the detectors modeled in (a) are modeled with a simulated dark count rate of 500 cps. The difference in performance at (d) the 15 mm source–detector separation and (e) the 30 mm source–detector separation show similar trends as shown in (a) and (b), with the few-mode fiber providing modest improvement in CNR over the single-mode fiber at both the short and long source–detector separation in the noiseless condition. The greater benefit of few-mode fiber is seen at the longer source–detector separation (e), where the decrease in CNR with increasing dark counts is reduced relative to the single-mode fiber.

Unlike for NIRS, where multimode fibers and fiber bundles are used, large increases in detector fiber diameter do not appreciably improve DCS signal quality.^53^ The increased intensity fluctuations measured on the detector due to each additional independent fiber mode are canceled out by the increase in the shot noise of the increased average intensity. However, this is only strictly true for noiseless systems. The contribution of read and dark noise to speckle measurements makes reaching shot-noise limited performance difficult when using single-mode fiber, and the use of larger fibers in DCS or smaller s/p ratio in SCOS can allow for the compensation of these real-world non-idealities. To demonstrate the improvement provided by the increased number of modes being measured by each detection element, we show the results of the expected CNR for DCS utilizing single-mode fiber and few-mode fiber in Fig. 6. For 850 nm, this is a comparison between two mode illumination in the case of the single-mode fiber, and 12 mode illumination in the case of the few-mode fiber. For 1064 nm, the single-mode fiber guides two modes, and for the same few-mode fiber (SMF-28), the fiber carries six modes at the longer wavelength. The results presented in Figs. 6(a) and 6(b) show the performance for a noiseless DCS detector with a single source at 850 and 1064 nm, respectively. For the noiseless detectors, as expected, there is a modest benefit in CNR with the use of few-mode fiber over single-mode fiber for both wavelengths. The benefit is seen to increase when realistic dark counts of the detector are considered. The larger benefit of the few-mode fiber is shown in Fig. 6(c).^41^ The range of dark/room light counts explored here are consistent with what is expected for a thick silicon SPAD^55^ or for a measurement done in a bright environment. For shorter separation measurements, the count rate is high enough that for the range of noise counts simulated, the effect on the contrast-to-noise ratio is negligible [Fig. 6(d)]. For longer separations though, the influence of noise counts becomes evident, and the use of few-mode fiber allows for nearly comparable performance to the noiseless case, even with an extreme dark count rate (for Si SPADs) of 1500 cps [Fig. 6(e)].

**Fig. 7:**
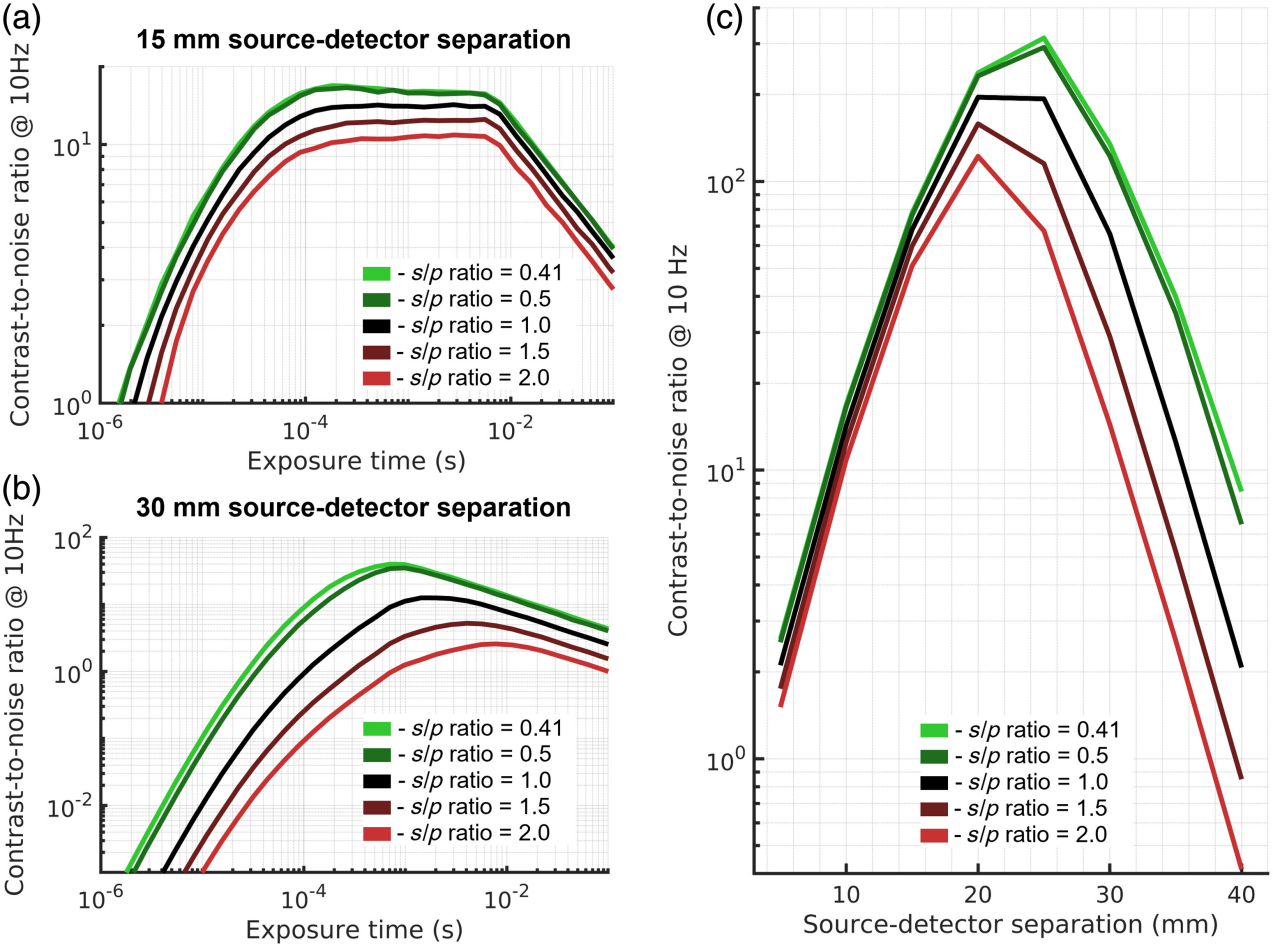
Comparison of SCOS CNR as a function of the s/p ratio. For both (a) short and (b) long source–detector separation, the use of a smaller s/p ratio achieves the highest CNR by allowing for more independent speckle observations as well as increasing the intensity measured by each pixel of the camera sensor, enabling shot noise to overwhelm the other sources of noise. (c) The results are compared as a function of source–detector separation, and the greatest benefit to decreasing the utilized s/p ratio is found for long source–detector separation measurements.

For SCOS, we explored a similar effect caused by the difference in the size of speckles projected onto the camera sensor. This effect has been explored previously in the literature,^45^ and here we expand the analysis to different source–detector separations and evaluate the contrast-to-noise ratio achieved through the use of different s/p ratios. The results presented here show the maximal possible CNR produced by either of the pulsed laser strategies, whether frame rate limiting or max power limiting. For short separation measurements, in a comparison of the contrast-to-noise ratio at 15 mm with different s/p ratios [Fig. 7(a)], the use of small s/p ratio projection allows for the use of shorter exposure times due to reaching shot noise limited performance more quickly, increasing the sensitivity to the cerebral signal, thus increasing CNR. In addition, when the number of speckles available is much greater than the number of pixels, for reductions in s/p ratio to s/p≈0.4,^45^ the number of independent speckle observations (NIOs) increases, improving the averaging characteristics, and SNR of the measurement. For s/p ratio<∼0.4, the number of independent observations is approximately equal to the number of pixels (for s/p=0.4, $\mathrm{NIO}=0.95*n_{\text{pixel}}$^45^) and further reductions of s/p ratio will only minimally affect the number of independent observations. Further, if the number of available speckles is not sufficient to utilize all pixels on the camera, an overall decrease in the SNR of the measurement will be observed for decreasing s/p ratio. For long separation measurements [Fig. 7(b)], the use of smaller s/p ratios allows for the measurement to reach shot-noise limited performance before the exposure time becomes long enough to affect frame averaging, maximizing possible CNR. We make the comparison of maximal achievable CNR as a function of source–detector separation and compare across s/p ratio values in Fig. 7(c), demonstrating the benefits given by projecting a smaller speckle size.

### 3.4 Effect of Extracerebral Thickness on the Simulated Performance of SCOS and DCS

**Fig. 8:**
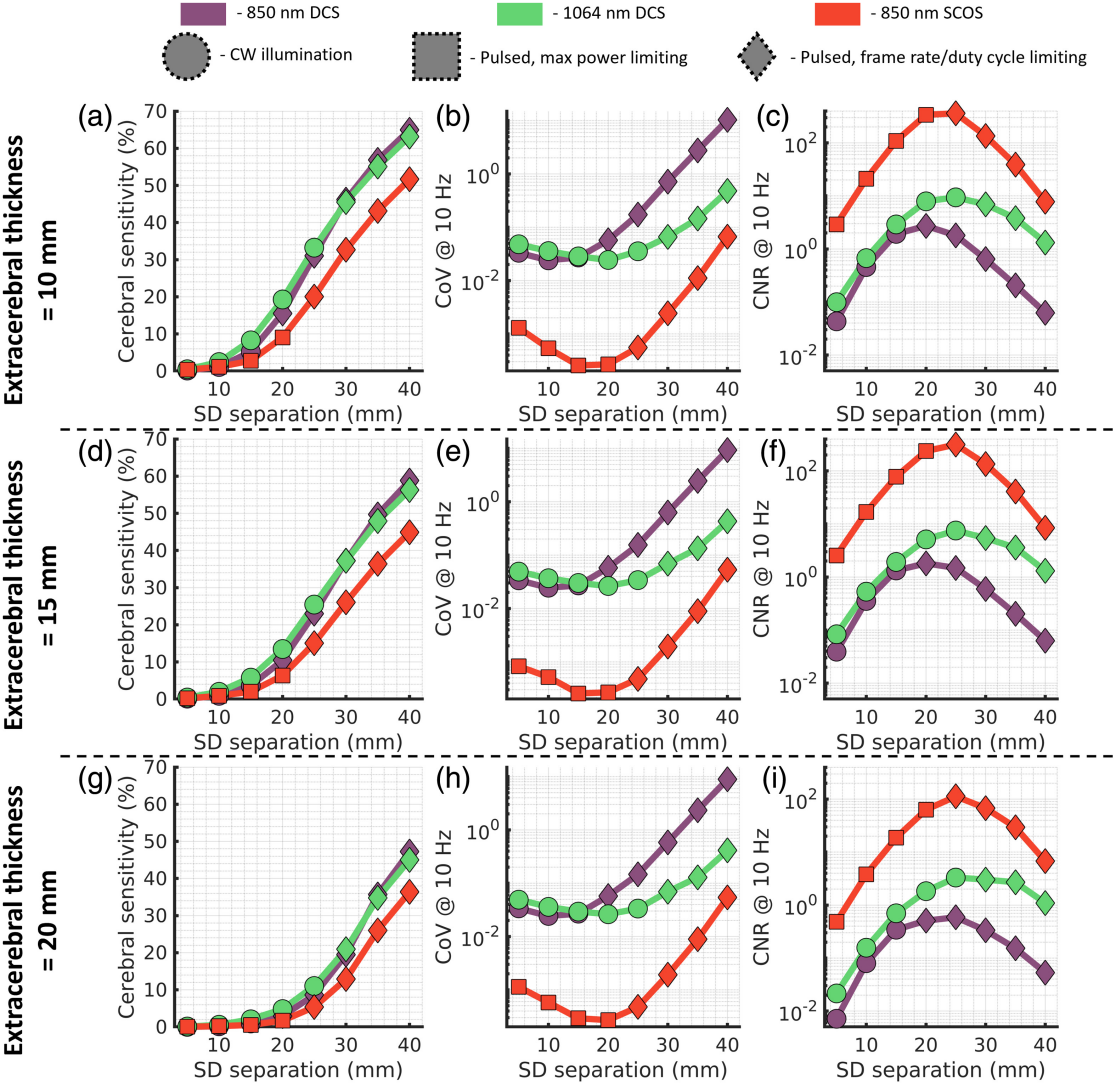
(a), (d), (g) Comparison of sensitivity, (b), (e), (h) CoV, and (c), (f), (i) contrast-to-noise ratio between the two DCS implementations and the one SCOS implementation across simulations with different extracerebral thicknesses. The CoV can be seen to not vary greatly between extracerebral thicknesses, with slight deviation in the SCOS shorter source–detector simulations. Cerebral sensitivity falls off with increasing extracerebral thickness, as was observed previously,^30^ and as was seen in Figs. 2(a) and 3(a), DCS exhibits slightly higher sensitivity at a given source–detector separation. Contrast-to-noise ratio decreases with increasing extracerebral thickness and is seen to be highest for 850 nm SCOS at any given source–detector separation considered. The scaling of the y-axis for each parameter explored (sensitivity, CoV, and CNR) is kept constant across extracerebral thicknesses to aid in comparison.

Following the exploration of experimental factors that we have control over, we examined the effect of subject specific geometric factors on the diffuse optical measurements. For each of the three explored extracerebral tissue thicknesses (10, 15, 20 mm), we present the cerebral sensitivity [Figs. 8(a), 8(d), and 8(g)], CoV [Figs. 8(b), 8(e), and 8(h)], and CNR [Figs. 8(c), 8(f), and 8(i)] for each of the three techniques explored here (850 nm DCS, 1064 nm DCS, and 850 nm SCOS). For DCS results, we present the optimal laser delivery strategy (either CW or duty cycle limiting) in terms of contrast-to-noise ratio for single-mode fiber. For SCOS results, we present the optimal pulsed laser strategy (max power limiting or frame rate limiting) for the smallest possible s/p ratio (s/p=0.41). Since it is often preferable to minimize optical probe size for practical reasons, we do not consider the multi-source approach for this analysis.

Overall, increasing extracerebral thickness decreases cerebral sensitivity and minimally affects CoV, leading to an overall reduction in CNR. While cerebral sensitivity is lower for SCOS at the optimal CNR operating point, the contrast-to-noise ratio is consistently higher than either DCS implementation across all explored source–detector separations and extracerebral thicknesses. The influence of increased extracerebral thickness on the sensitivity is exaggerated at shorter source–detector separations as compared to longer separations, which shifts the peak of the CNR curve to longer source–detector separations with increasing extracerebral thickness. We also label the optimal laser source strategy used for each source–detector separation and extracerebral thickness. For DCS, the switch from CW operation to pulsed operation happens at commonly used source–detector separations (15 to >20  mm for 850 nm, 25 to >30  mm for 1064 nm).

## 4 Discussion and Conclusion

**Fig. 9:**
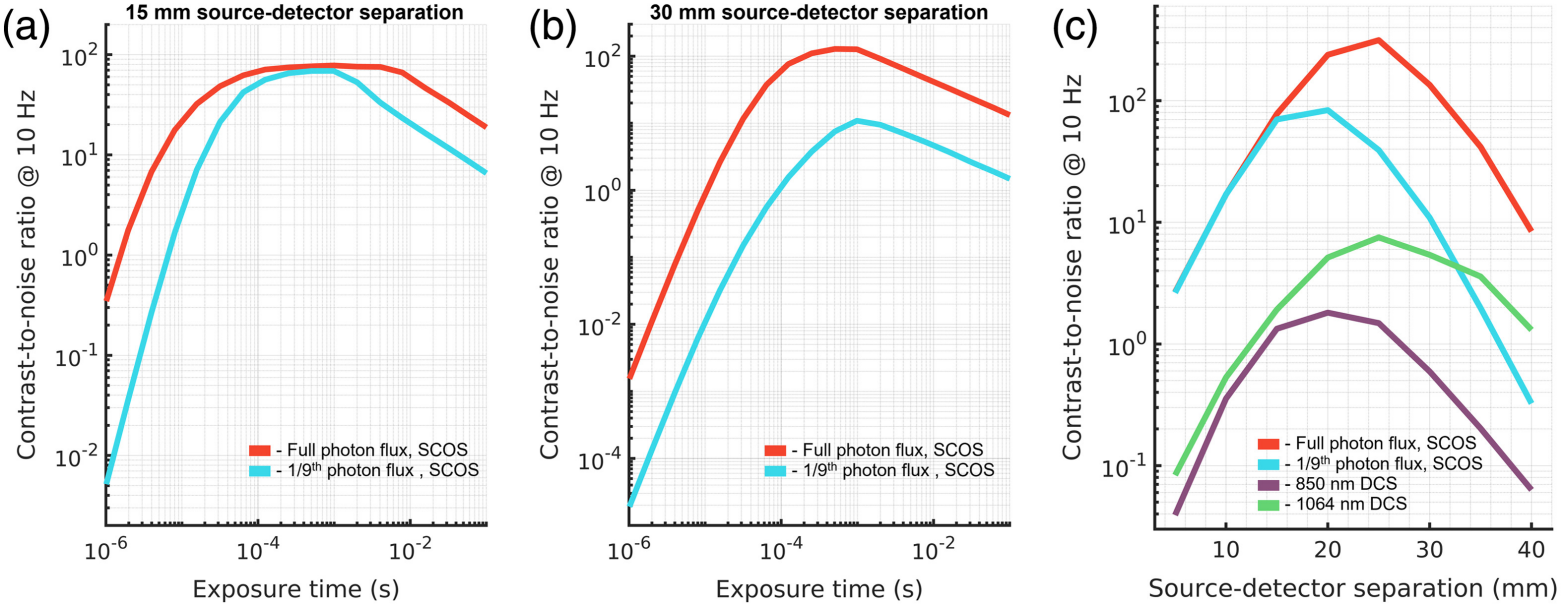
Comparison of simulated SCOS performance for (a) short and (b) long source–detector separation as a function of exposure time, and (c) as a function of source–detector separation at optimal exposure time. The performance at short separation is minimally affected by the reduced per mode photon flux, though at long separation, the reduction in contrast-to-noise ratio is ∼15×.

In this work, we have evaluated the potential performance for two implementations of DCS and one implementation of SCOS for the measurement of CBF using Monte Carlo simulations of subject specific MRI models and appropriate signal noise models. We have observed the large possible improvements in SNR and contrast-to-noise ratio offered by SCOS. The somewhat lower SCOS sensitivity to the cerebral signal as compared to DCS at the optimal CNR operating condition could be due to the single exposure measurements explored, which inherently reduces the information collected by the measurement, as well as using an exposure time much greater than the decorrelation time of the autocorrelation function. Previous work has demonstrated reconstruction of DCS such as data from SCOS speckle contrast measured at multiple exposures, which crucially include exposure times on the order of the decorrelation time where the cerebral sensitivity of single exposure SCOS is comparable to the cerebral sensitivity of DCS [Figs. 2(a) and 3(a)]. This approach would allow for the same cerebral sensitivity to be achieved.^62^ In practice, the reduced SNR of the shorter exposure time measurements, as well as the need to sacrifice frame averaging to collect multiple exposures is more detrimental to the CoV, and the slight decrease in sensitivity of the single exposure measurement is more than made up for with the massively parallelized speckle measurements, reflected in the almost two orders of magnitude estimated improvement in maximally achievable CNR. While this simulation study points to SCOS as a superior technique for the measurement of CBF, several simplifying assumptions are made that may limit what is achievable in practice. As was noted in previous work,^63^ in MRI scans, hair is invisible, though has a great influence on the detectability of light in optical experiments (∼20% to 50% reduced photon flux).^64^ While we ignored the effects of hair in this work, the reduction in SNR caused by the presence of hair should affect both DCS and SCOS similarly. For both SCOS and DCS, we assume here that saturation of the detector is not possible, therefore allowing unrealistically high signal levels to be achieved. This affects SCOS and DCS in different ways. For DCS, implementing the true hold-off time of the single photon detectors will cause distortions of the correlation function as the count rate reaches closer to the maximal possible count rate ($1/\tau_{\text{Hold off}}$). For SCOS, saturation of the detector would artificially decrease the measured contrast by clipping the upper levels of the signal and would negatively affect both the SNR and accuracy of the measurement. For both techniques, this largely affects shorter source–detector separations where photon flux is high relative to the maximum count rate of the detector and likely does not greatly affect the estimates at longer source detector separations, which are already in a photon-starved regime. In addition, for SCOS, projection of light onto the simulated imaging detector was assumed to be uniform, which may be difficult to achieve in practice due to the different sizes and shapes of fiber bundles and imaging detectors. Further, a recent work showing very strong results with SCOS for *in vivo* measurement of CBF^18^ noted that the throughput in the SCOS system per-fiber mode utilized was ∼9× lower than the per-fiber mode throughput of DCS. Optical coupling to the detector array has large implications on the possible performance of SCOS and is not considered in this work. To examine the above effect in this model implementation, we have simulated an SCOS system with 9× lower photon flux per mode with the optimal laser pulsing strategy and show the results in Fig. 9. We include the optimal CNR of 850 and 1064 nm DCS in Fig. 9(c) for comparison.

This discrepancy between theoretical performance and realistic performance is dependent on the selection of electrical and optical components that make up a particular system. For the simulated DCS systems explored here, we selected a commonly used silicon SPAD detector to explore what is realistically expected in current DCS systems. For 1064 nm DCS, we used an SNSPD that allows for optimal measurements and has started to emerge in the DCS field.^42^^,^^65^^,^^66^ With a great range of possible imaging detectors, after surveying several possible camera detectors, we aggregated the properties into a CMOS camera with average values for read noise, number of pixels, frame rate, and quantum efficiency. The selection of these properties has great influence on the outputs of the model. As an example, the reduced photon flux explored in Fig. 9 could be offset by selecting a camera with a higher quantum efficiency. This highlights the importance of the selection of the detection chain, which includes the fiber bundle, relay optics, and the image sensor. In general, we expect these results to reflect the expected performance of the techniques explored, and the insights gained through the evaluation of these three implementations are likely generalizable to a greater range of systems. For longer separation DCS measurements, the use of a pulsed laser strategy could improve performance without the increased bulk and spatial resolution impacts of a multi-source design. The use of the pulsing strategy is essential to elevate the performance of SCOS, as optical losses through the system could reduce the instantaneous photon flux to a detrimental level for long source–detector separation measurements. Further, although single channel measurements were considered in this work, the laser pulsing strategies are beneficial to multi-channel systems employed for tomographic imaging as well. Due to the opportunity for temporal multiplexing of spatial emission positions, for speckle-based imaging, the use of a pulsed strategy can allow for improved photon flux across many channels. In summary, for SCOS improving photon flux per mode will fully exploit the potential of this technology to achieve high performance with low-cost detectors.

In some situations, with the recent availability of SPAD arrays,^9^^–^^11^ both SCOS and DCS measurements could be made with the same detection hardware and the decision as to which method will maximize CNR for the CBF signal needs to be made. In a simulation with matched per mode photon flux, quantum efficiency, read noise ($0\;e^{-}$), and pixel number, we found that on a per pixel basis, DCS provides a greater CNR (∼10× when the optimal laser pulsing strategy is used) than SCOS across all source–detector separations and would be the preferred technique for the measurement of CBF in this particular situation (results not shown).

A separate question that could form the subject of future work is what CNR level is needed for accurate *in vivo* measurements, which are impacted by physiological noise sources—in a sense, what is needed to achieve physiological noise limited measurements. While out of the scope of this paper, it is important to keep the influence of natural biological fluctuations in mind when comparing pure instrument noise performance, as is done in this work. Further, the simulations presented here do not include an exploration of the impacts of extracerebral physiology cross-talk. This may be less impactful for functional measurements with well demarcated functional stimuli, as the techniques to address extracerebral contamination in these controlled settings are well-established.^46^ For more naturalistic stimuli or clinical blood flow measurements, the impact of superficial signal contamination may degrade the contrast-to-noise ratio differently for DCS and SCOS given that they have different levels of extracerebral sensitivity. Finally, other practical aspects may impact optimal operating points—for example, DCS measurements allow continuous tracking of the coherence factor β, which is proportional to the fraction of coherent photons among the overall photon flux detected. The value of β can fluctuate if environmental conditions change. For SCOS to compensate, a multiple-exposure time strategy may be needed, at least intermittently, which would reduce the benefits of frame averaging by the square root of the number of unique exposures.

To conclude, we hope these simulations can serve as a guide for optimizing the experimental properties for DCS and SCOS measurements. Realistic operating conditions and other factors as discussed above should also be taken into consideration when planning optical blood flow monitoring studies.

## Data Availability

A library of scripts used to compute the metrics presented in the work are publicly available as an example in the scatterBrains repository. Data supporting the results reported in the manuscript may be requested by contacting the corresponding author.
