# Supplementary material for: Comparing the performance potential of speckle contrast optical spectroscopy and diffuse correlation spectroscopy for cerebral blood flow monitoring using Monte Carlo simulations in realistic head geometries
Source: Neurophotonics. 2024 Jan 27;11(1):015004. doi: 10.1117/1.NPh.11.1.015004 (PMC10821780; doi:10.1117/1.NPh.11.1.015004)
Supplement: Supplementary file 1 [file NPh_011_015004_SD001.pdf]

Supplement to comparing the performance potential of speckle contrast optical spectroscopy (SCOS) and diffuse correlation spectroscopy (DCS) for cerebral blood flow monitoring using Monte Carlo simulations in realistic head geometries

Mitchell B. Robinson,[a,*] Tom Y. Cheng,[a,b] Marco Renna,[a] Melissa M. Wu,[c] Byungchan Kim,[b] Xiaojun Cheng,[b] David A. Boas,[b] Maria Angela Franceschini,[a] Stefan A. Carp[a]
[a]Athinoula A. Martinos Center for Biomedical Imaging, Department of Radiology, Massachusetts General Hospital, Harvard Medical School, MA 02129, USA
[b]Neurophotonics Center, Department of Biomedical Engineering, Boston University, MA 02215, USA
[c]Department of Biomedical Engineering, Duke University, NC 27708, USA

S1. Example of typical dark noise for silicon CMOS camera

Because a large range of exposure times are explored in this work (1 μs to 100 ms), the assumption for the model neglecting the contributions of dark noise may be violated. To explore this experimentally, we collected dark frames using a silicon CMOS camera (Basler ace acA1300-200um) at a range of exposure times and computed the average intensity collected as well as the combination of dark and read noise variance. Average intensity and variance are computed on windows of size 8 pixels by 8 pixels with 0% overlap, and the average and variance values for each window are averaged across multiple collected dark images (20 images per exposure time). Shown below in Figure S1, the average intensity (Figure. S1.a) and combined noise variance (Figure S1.b) distributions are relatively flat for much of the exposure time range and are only found to elevate at exposure times >~25 ms. While this effect would be noticeable for the longer exposures and would result in an increased coefficient of variation, for the simulations performed with the described camera settings, CoV estimates for exposure times >6.67 ms already increase due to the loss of frame averaging. Additionally, in practice, the average dark frame and dark variance at the long exposure would be subtracted, accounted for the longer exposure. The optimal CNR operating point for most measurements presented occurs at an exposure time shorter than 25

ms, and although the assumption does break down for the longer exposure range, it should not have a large effect on the selection of optimal operating parameters.

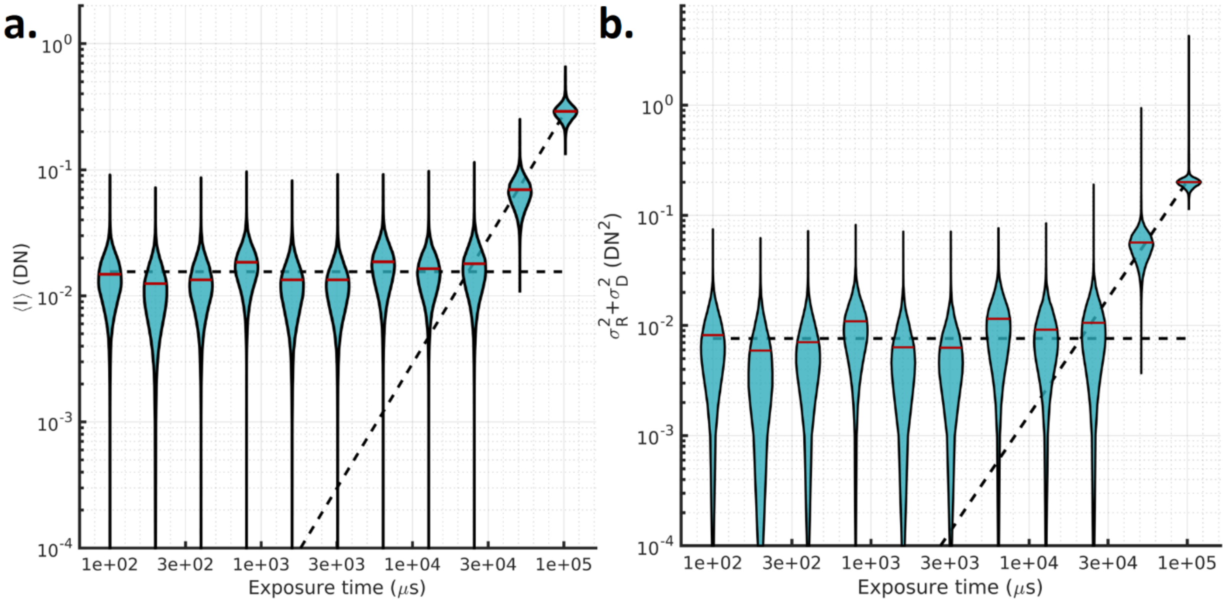

Figure S1. Comparison of the dark intensity (a.) and the dark variance (b.) measured as a function of exposure time on a representative silicon CMOS camera. For both average intensity and variance, increases in the parameter values are not observed until exposure times >~25ms. These results would indicate for a reasonably similar camera, measurements with exposure times less than ~25ms will be relatively unaffected by the influence of dark current, and the assumption is reasonably valid for the vast majority of optimal operating parameters explored in the main text.

S2. Sensitivity to changes in cerebral blood flow (CBF) as a function of the relative change in CBF.

In this work, a cerebral blood flow perturbation of 20% was selected to evaluate the cerebral sensitivity of the SCOS and DCS measurements. This CBF change is consistent with changes we have observed in previous studies in response to both functional activation and physiological manipulations[14,31]. While it is generally assumed that sensitivity to the cerebral signal is relatively constant within the physiologic range of blood flow values, we investigate the effect of changing the magnitude of the CBF perturbation on the extracted BF_i and the sensitivity of the measurement. In Figure S2, the comparison of absolute cerebral sensitivity (Fig. S2.a and S2.c) and difference in

cerebral sensitivity relative to the cerebral sensitivity computed for a 10% change in CBF (Fig S2.b and S2.d) are shown for both DCS and SCOS for a range of CBF changes from 10% to 100%. From the results, cerebral sensitivity of DCS is less affected by the change in magnitude of the perturbation as compared to the cerebral sensitivity of SCOS, though the sensitivities found have a relatively small range across the range of changes in CBF. These results may also suggest shortcomings of the semi-infinite model applied to multi-layered tissue, as previous studies using multi-layered models have demonstrated accurate recovery of relative changes in CBF[33].

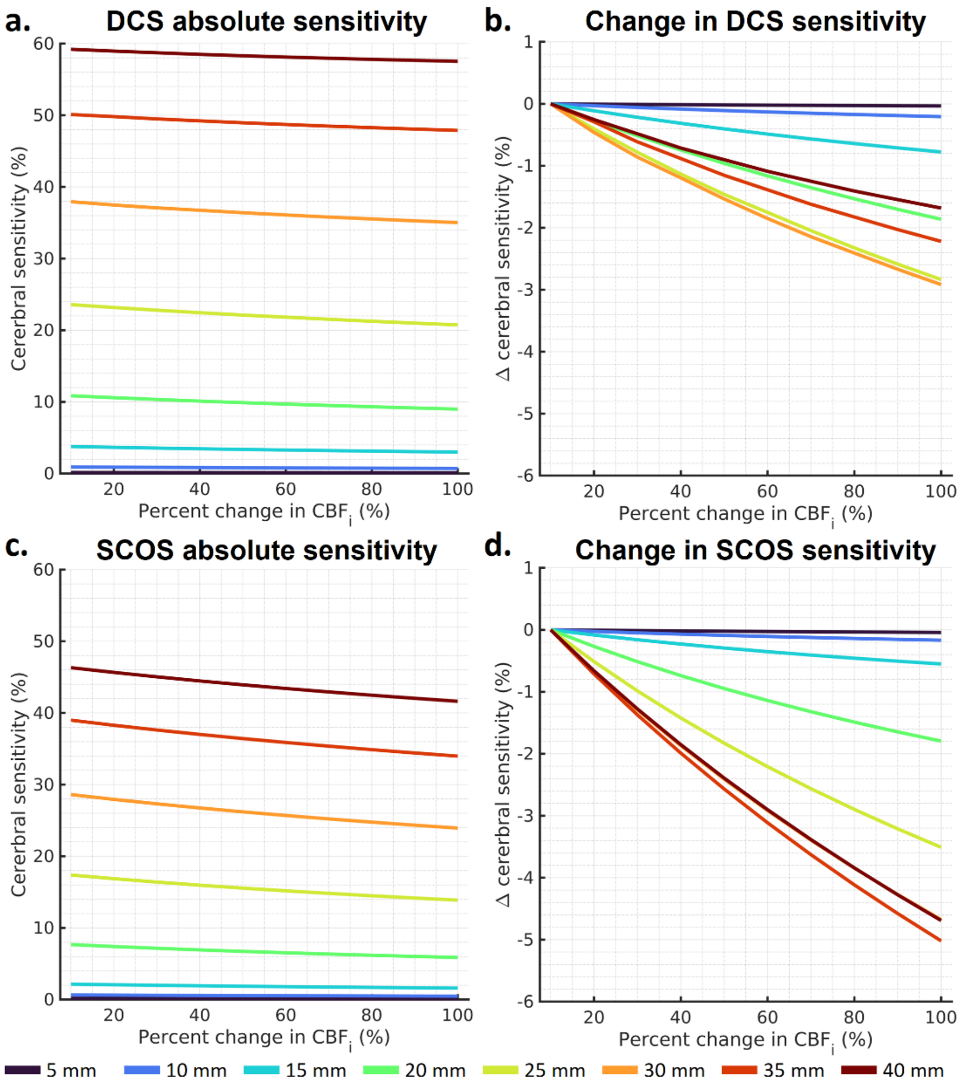

Figure S2. Comparison of the estimated cerebral sensitivity for both DCS (a.) and SCOS (c.). The change in estimated cerebral sensitivity as a function of perturbation magnitude is also shown for both DCS (b.) and SCOS

(d.). While there are differences across the perturbation magnitude range, the findings here would not appreciably affect the results presented in the main text.

S3. Expanded description of the regimes present in the SCOS contrast-to-noise curves at different exposure times:

For the different laser illumination strategies explored, the shape of the SCOS contrast-to-noise curve is relatively complicated, and the shapes reveal the interplay between the exposure time, max frame rate, maximum laser power available, the ANSI limited source power, and the noise properties of the camera. In Figure 5 in the main text, we explored four laser illumination strategies: 1) single CW source, 2) multiple CW sources to utilize the entire max laser power without breaking the ANSI limit, 3) a pulse width modulated strategy where the source input power (P_{in}) is modulated such that the product of the input power, exposure time (T_{exp}), and the frame rate of the camera (f_s) is less than or equal to the ANSI limited power (P_{ANSI}) for a 3.5 mm diameter, single-source position, expressed as $P_{in} * f_s * T_{exp} \leq P_{ANSI}$, and 4) a pulse width modulated strategy where the frame rate of the camera is modulated such that the product of the maximal input power (P_{max}), exposure time and the frame rate of the camera is less than or equal to the ANSI limited power for a 3.5 mm diameter, single-source position, expressed as $P_{max} * f_s * T_{exp} \leq P_{ANSI}$. These figures reveal three operation regimes for SCOS, which affect each of the different illumination styles in different ways. Shown in Figure S3, we expand upon the figures shown in the main text and detail the input power used for each strategy at a given exposure time (Figure S3.a), the frame rate utilized for each exposure time (Figure S3.b), the duty cycle of the measurement (Figure S3.c), and a figure depicting the data shown in Figure 5.b with shading to demarcate the transition from one regime of operation to another. In the region labeled (1) in Figure S3.d, the simulated measurement has yet to reach the point of being ANSI limited for the pulsed configurations. In this region the CNR for all source delivery strategies exhibits a monotonic

increase with exposure time, indicating the measurements are not yet shot noise limited. At the boundary of the region labeled (1) and the region labeled (2), the power delivery for the pulsed laser strategies reaches the ANSI limit, given by $P_{max} * f_{max} * T_{exp} = P_{ANSI}$, and marks the first point of divergence between the different implementations. The selection of either reducing the input power (Figure S3.a) or reducing the frame rate (Figure S3.b) results in a different shape in the CNR curve. As was noted in the main text, for long separation measurements, increasing the instantaneous photon flux during the exposure time is more beneficial for the measurement CNR, as seen in Figure S3.d. Further, for measurements made with sufficiently small s/p ratio to reach shot noise limited performance, for the simulations performed in this work of the pulsed laser strategies, the exposure time equal to $\frac{P_{ANSI}}{P_{max} * f_{max}}$ provides optimal performance. The boundary between the region labeled (2) and the region labeled (3) occurs at the exposure time equal to the inverse of the max frame rate and represents the point at which camera frame rate and the degree of frame averaging will be reduced by the increased exposure time. For most measurement conditions, a monotonic decrease in CNR is seen for all source configurations after this exposure time, though there are cases, as seen in Figure 7.b (s/p ratio = 2.0), where the peak of the CNR curve is reached after the frame rate is limited by the exposure time. This difference represents a condition in which the measurement is not shot noise limited and the increase in the number of collected photons outweighs the reduction in frame averaging.

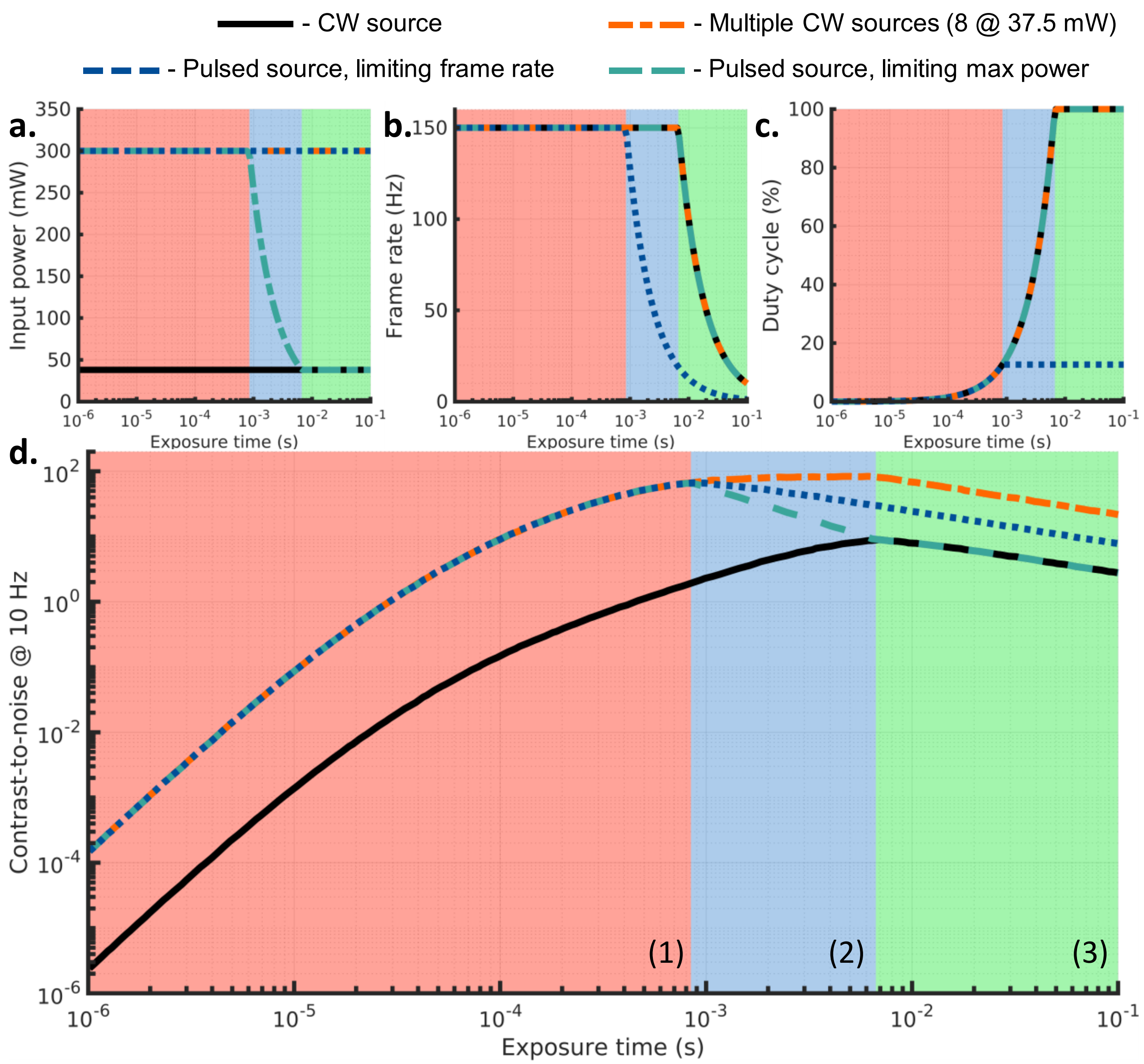

Figure S3. Expanded description of the factors that differentiate the different laser pulsing strategies as well as describe the different regimes of the SCOS CNR curve. In (a.) the input power used for each strategy is detailed. Both pulsed source implementations match the multi-source CW input power until the boundary between regions (1) and (2), after which the max power limiting strategy approaches the single CW source approach. In (b.) the frame rate used for each strategy is detailed. The reduction in frame rate from the frame rate limiting strategy can be seen after the boundary between regions (1) and (2), and the monotonic decrease continues with increasing exposure time to maintain a duty cycle equal to the ratio of the ANSI-limited single source power and the max power of the laser, as seen in (c.). For the other laser strategies, the duty cycle monotonically increases until reaching region (3), when the exposure time limits the frame rate of the measurement, and the duty cycle reaches 100%. The results presented in Figure 5.b are presented again in (d.) with additional labeling describing the transitions between different regions of the curve. The boundary between regions (1) and (2) occurs at an exposure time equal to $\frac{P_{ANSI}}{P_{max}*f_{max}}$, and the boundary between regions (2) and (3) occurs at an exposure time equal to $\frac{1}{f_{max}}$.

S4. Description of the simulation pipeline from Monte Carlo simulations to fitting for blood flow index

As a reference, we have included a full graphical description (Figure S4) of the simulation pipeline to demonstrate the different steps required to reproduce the results shown in the main text. The description detailed in the main text is summarized into a branched six step process which includes: (1) Monte Carlo simulation of light propagation through the realistic tissue volume, collecting information about the dimensionless momentum transfer, partial pathlength in each tissue type, and diffuse reflectance intensity profile[32]; (2) Calculation of the electric field autocorrelation functions $(g_1(\rho,\tau))$ for each source detector separations are performed for both the baseline and perturbed states using the outputs of the Monte Carlo model[24]; (3) Calculation of the expected number of photons per mode per second for each source-detector separation given the source illumination strategy and the Monte Carlo derived detected intensity normalized by a previously reported reference measurement[42]; (4a & 4b) Calculation of the expected number of photons per second incident on each detector, which depends on the quantum efficiency of the detector and the number of modes projected onto the detector, and the coherence parameter, which depends on the number of independent modes incident on the detector; (5a & 5b) Generation of the noisy data that is fit for blood flow index using the appropriate signal models. For DCS, the Siegert relation[21] is used to convert the simulated electric field autocorrelation function to the intensity autocorrelation function, and noise is added using the previously described DCS noise model[43,44]. For SCOS, the squared speckle contrast is computed from the simulated electric field autocorrelation function, and the noise of the squared contrast is computed using the previously described SCOS noise model[45]; (6a & 6b) Fitting of the noisy data is done using the solution to the correlation diffusion equation for a semi-infinite media in the reflectance geometry.

1

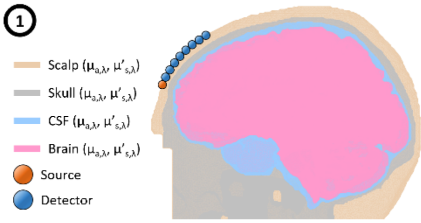

Monte Carlo simulation outputs: matrix of photon partial pathlengths ($\boldsymbol{L_\rho}$) and momentum transfers ($\boldsymbol{Y_\rho}$) for each source-detector separation (ρ) for all photon trajectories detected by that detector ($N_{p,\rho}$) for each of the tissue types (N_t) in addition to the diffuse reflectance distribution.

$$\boldsymbol{L_\rho} = \begin{bmatrix} L_{\rho,1,1} & L_{\rho,2,1} & \cdots & L_{\rho,N_{t,\rho},1} \\ L_{\rho,1,2} & L_{\rho,2,2} & \cdots & L_{\rho,N_{t,\rho},2} \\ L_{\rho,1,3} & L_{\rho,2,3} & \cdots & L_{\rho,N_{t,\rho},3} \\ L_{\rho,1,4} & L_{\rho,2,4} & \cdots & L_{\rho,N_{t,\rho},4} \end{bmatrix} \quad \boldsymbol{Y_\rho} = \begin{bmatrix} Y_{\rho,1,1} & Y_{\rho,2,1} & \cdots & Y_{\rho,N_{t,\rho},1} \\ Y_{\rho,1,2} & Y_{\rho,2,2} & \cdots & Y_{\rho,N_{t,\rho},2} \\ Y_{\rho,1,3} & Y_{\rho,2,3} & \cdots & Y_{\rho,N_{t,\rho},3} \\ Y_{\rho,1,4} & Y_{\rho,2,4} & \cdots & Y_{\rho,N_{t,\rho},4} \end{bmatrix}$$

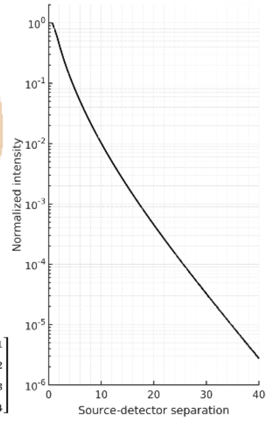

2

Calculation of electric field autocorrelation functions for the baseline and perturbed condition. Correlation function data is a matrix of values of size $[N_\rho, N_\tau, 2]$.

$$g_1(\rho,\tau) = \frac{\sum_{n=1}^{N_{p,\rho}} \exp\left(-\frac{1}{3}k_0^2 \sum_{i=1}^{N_t} Y_{\rho,n,i}\langle\Delta r^2(\tau)\rangle_i\right)\exp\left(-\sum_{i=1}^{N_t}\mu_{a,i}L_{\rho,n,i}\right)}{\sum_{n=1}^{N_{p,\rho}}\exp\left(-\sum_{i=1}^{N_t}\mu_{a,i}L_{\rho,n,i}\right)}$$

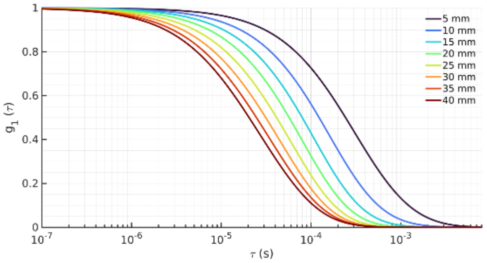

3

Calculation of photons per mode per second ($\langle I(\rho)\rangle_{mode}$) given the laser strategy used and the appropriate power scaling factor (f_{scale}), the reference photons per mode for an ANSI limited source $\left(I_{ref}(\rho_{ref})\right)$ at a reference source-detector separation (ρ_{ref}), the normalized Monte Carlo diffuse reflectance curve $(I_{MC}(\rho))$, and the source-detector separation (ρ).

$$\langle I(\rho)\rangle_{mode} = f_{scale}\frac{I_{ref}(\rho_{ref})}{I_{MC}(\rho_{ref})}I_{MC}(\rho)$$

DCS

SCOS

4a

Calculation of DCS beta value (β_{DCS}) and photons per detector per second ($\langle I(\rho)\rangle_{det}$) based on the polarization of the detected light (N_{pol}), the number of spatial fiber modes (M), and the detector quantum efficiency (QE).

$$\langle I(\rho)\rangle_{det} = (N_{pol}\cdot M\cdot QE)\,\langle I(\rho)\rangle_{mode} \qquad \beta_{DCS} = \frac{1}{N_{pol}}\frac{1}{M}$$

4b

Calculation of SCOS beta value (β_{SCOS}) and photons per detector per second ($\langle I(\rho)\rangle_{det}$) based on the polarization of the detected light (N_{pol}), the s/p ratio and the quantum efficiency (QE).

$$\langle I(\rho)\rangle_{det} = \left(QE\frac{N_{pol}}{(^s/_p)^2}\right)\langle I(\rho)\rangle_{mode} \qquad \beta_{SCOS} = \frac{1}{N_{pol}}\frac{(^s/_p)^2}{1+(^s/_p)^2}$$

5a

Calculation of expected DCS noise $(\sigma_{DCS}(\tau,\rho))$ given the sampling rate of the measurement (f_s), the time step of the correlation function time lags (T), the photons per detector, the duty cycle for a particular laser strategy (α_{DC}), the DCS beta value, and the estimated single exponential decay rate of $g_2(\rho,\tau)$ $(\Gamma(\rho))$.

$$\sigma_{DCS}(\tau,\rho) = \sqrt{\frac{Tf_s}{\alpha_{DC}}}\left[\begin{matrix}\beta_{DCS}^2\frac{\left(1+e^{-2\Gamma(\rho)T}\right)\left(1+e^{-2\Gamma(\rho)\tau}\right)+2\frac{\tau}{T}\left(1-e^{-2\Gamma(\rho)T}\right)e^{-2\Gamma(\rho)\tau}}{1-e^{-2\Gamma(\rho)T}}+.. \\ 2(T\cdot\langle I(\rho)\rangle_{det})^{-1}\beta_{DCS}\left(1+e^{-2\Gamma(\rho)\tau}\right)+.. \\ (T\cdot\langle I(\rho)\rangle_{det})^{-2}\left(1+\beta_{DCS}e^{-\Gamma(\rho)\tau}\right)\end{matrix}\right]$$

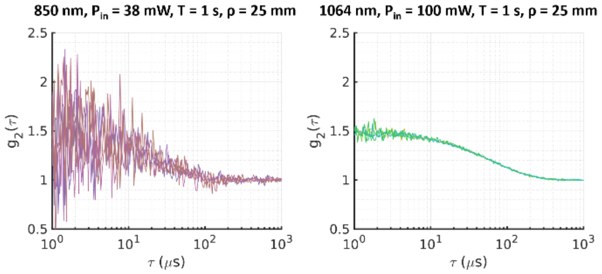

Multiple examples of the simulated autocorrelation function noise for DCS measurements at 850 nm and 1064 nm with the same source detector separation ($\rho = 25\ mm$), and integration time ($T = 1\ s$). The increased photon flux at 1064 nm (~10x) can be seen to greatly improve the SNR of the autocorrelation functions.

5b

Calculation of both the speckle contrast contributions from read noise $(K_r^2(\rho))$, shot noise $(K_s^2(\rho))$, and fundamental speckle contrast $(K_f^2(\rho))$, as well as the noise contributions of each term $\left(\sigma_{K_r^2},\sigma_{K_s^2},\sigma_{K_f^2}\right)$ given the electric field temporal autocorrelation function, exposure time (T_{exp}), number of photons per detector, SCOS beta value, read noise variance of the camera (σ_r^2), number of pixels (n_{pixel}), and number of independent speckles measured (NIO).

$$K_f^2(\rho) = \frac{2\beta_{SCOS}}{T_{exp}}\int_0^{T_{exp}} g_1^2(\rho,\tau)\left(1-\frac{\tau}{T_{exp}}\right)d\tau \qquad \sigma\left(K_f^2(\rho)\right) = K_f^2(\rho)\sqrt{\frac{2\left(1+2K_f^2(\rho)\right)}{NIO}}$$

$$K_s^2(\rho) = \frac{1}{\langle I(\rho)\rangle_{det}T_{exp}} \qquad NIO = \frac{n_{pixel}}{1+0.292(^s/_p)^2} \qquad \sigma\left(K_s^2(\rho)\right) = 1.90\cdot K_s^2(\rho)\sqrt{\frac{1}{n_{pixel}}}$$

$$K_r^2(\rho) = \frac{\sigma_r^2}{\left(\langle I(\rho)\rangle_{det}T_{exp}\right)^2} \qquad \sigma\left(K_r^2(\rho)\right) = 1.47\cdot K_r^2(\rho)\sqrt{\frac{1}{n_{pixel}}}$$

$$\sigma(K^2) = \sqrt{\sigma^2\left(K_f^2\right)+\sigma^2(K_s^2)+\sigma^2(K_r^2)}$$

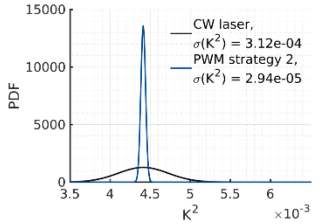

Comparison in the distribution of noisy K_f^2 values measured at 25 mm between CW illumination and pulsed illumination (strategy #4) not including frame averaging.

6a

Noisy intensity autocorrelation functions $(g_2(\tau))$are fit for BF_i using the solution to the correlation diffusion equation for a semi-infinite media in the reflectance geometry.

$$g_2(\tau) = 1+\beta_{DCS}|g_1(\tau)|^2$$

$$g_1(\tau) = \frac{r_b\exp(-K_{DCS}(\tau)r_1)-r_1\exp(-K_{DCS}(\tau)r_b)}{r_b\exp(-\sqrt{3\mu_a\mu_s'}r_1)-r_1\exp(-\sqrt{3\mu_a\mu_s'}r_b)} \qquad K_{DCS}(\tau) = \sqrt{3\mu_a\mu_s'+6k_0^2n^2\mu_s'^2BF_i\tau}$$

$$r_1 = \sqrt{\rho^2+1/\mu_s'^2} \qquad r_b = \sqrt{\rho^2+(1/\mu_s'+2z_b)^2} \qquad z_b = \frac{2}{3\mu_s'}\frac{(1+R_{eff})}{(1-R_{eff})}$$

$$R_{eff} = -1.44n^{-2}+0.71n^{-1}+0.668+0.0636n$$

6b

Noisy squared speckle contrast values are fit for BF_i inverting the speckle contrast integral with $g_1(\tau)$ taking the form of the solution to the correlation diffusion equation for a semi-infinite media in the reflectance geometry.

$$\min_{BF_i}\left(K^2-\widehat{K_f^2}\right)^2 \text{ where } \widehat{K_f^2} = \frac{2\beta_{SCOS}}{T_{exp}}\int_0^{T_{exp}} g_1^2(\rho,\tau)\left(1-\frac{\tau}{T_{exp}}\right)d\tau$$

Figure S4. Visual depiction of the simulation steps required to convert the outputs of the Monte Carlo simulations into estimates of blood flow index. Each step depicted in the figure is performed for each of the probe positions explored in this work. For each source-detector separation at each probe position, the partial pathlength in each tissue type and the dimensionless momentum transfer accrued over detected photon trajectories is saved (1). Additionally, the profile of diffuse reflectance at each probe position is computed to scale the expected photon flux at each detector. Using the Monte Carlo outputs, electric field autocorrelation functions $\left(g_1(\tau)\right)$ are computed (2). The photon flux per fiber mode per second is estimated using the illumination strategy and the scaled diffuse reflectance curve (3). For DCS and SCOS, the appropriate model for scaling the per mode photon flux is applied to calculate the per detector photon flux, and the appropriate model for the coherence parameter is used to calculate a technique specific β value (4). For each method, the appropriate signal and noise model is applied, and multiple instances of noisy data for each investigated condition are generated (5). Finally, the noisy data are fit for the blood flow index using the solution to the correlation diffusion equation for a semi-infinite media in the reflectance geometry and the appropriate fitting model. In the case of DCS, the Siegert relation is used, and in the case of SCOS, inversion of the speckle contrast integral is used (6).
